# Comparative metabolic profiling and quantitative analysis of metabolites in different tissues of *Ajuga turkestanica* by ESI-UHPLC-QqTOF-MS and NMR

**DOI:** 10.1038/s41598-024-71546-5

**Published:** 2024-11-15

**Authors:** Nilufar Z. Mamadalieva, Michal Šoral, Elana Kysil, Pauline Stark, Andrej Frolov, Ludger A. Wessjohann

**Affiliations:** 1 New Uzbekistan University, Movarounnahr Str 1, Mirzo-Ulug’bek District, 100000 Tashkent, Uzbekistan; 2 Institute of the Chemistry of Plant Substances, Uzbekistan Academy of Sciences, M. Ulugbek Str 77, 100170 Tashkent, Uzbekistan; 3 Tashkent Institute of Irrigation and Agricultural Mechanization Engineers, National Research University, Kori Niyazov Str. 39, 100000 Tashkent, Uzbekistan; 4 Department of Bioorganic Chemistry, Leibniz Institute of Plant Biochemistry, Weinberg 3, 06120 Halle, Germany; 5 Analytical Department, Institute of Chemistry, Slovak Academy of Sciences, Dúbravská cesta 9, SK-845 38 Bratislava, Slovak Republic

**Keywords:** *Ajuga turkestanica*, UHPLC-MS, NMR, Metabolite profiling, Metabolite annotation, Biological activity, Mass spectrometry, NMR spectroscopy, Structure elucidation

## Abstract

*Ajuga turkestanica* preparations are used as anti-aging cosmeceuticals and for medicinal purposes. Herein we describe the characterization and quantification of its metabolites in different organs using UHPLC-MS and NMR spectroscopy. A total of 51 compounds belonging to various phytochemical classes (11 flavonoids, 10 ecdysteroids, 9 diterpenes, 6 fatty acids, 5 iridoids, 3 phenylpropanoids, 3 sugars, 2 phenolics, 1 coumarin, 1 triterpene) were annotated and tentatively identified by UHPLC-ESI-QqTOF-MS/MS of methanolic extracts obtained separately from the organs. 1D and 2D NMR spectroscopy independently confirmed the identity of six major compounds. The abundances of these main constituents in flowers, fruits, leaves, roots, seeds, and stems were compared and quantified using ^1^H NMR. The results showed that 8-*O*-acetylharpagide, 20-hydroxyecdysone (ecdysterone) and ajugachin B were the most abundant constituents in the species. The two major compounds, 8-*O*-acetylharpagide and 20-hydroxyecdysone, were chosen as the markers for the quality assessment of *A. turkestanica* material. The methanolic extract of the aerial parts of *A. turkestanica* showed no noteworthy anthelmintic (antihelmintic), antifungal, or cytotoxic effect in in vitro assays.

## Introduction

Lamiaceae is a widespread family of flowering plants, also known as the mint family. Within this family, the genus *Ajuga* L. (Ajugeae tribe) is herbaceous and possesses annual or perennial forms, comprising around 90 species found in Europe, Asia, Africa, and Australia^1^. The species from *Ajuga* are widely employed as anthelmintic, astringent, febrifuge, diuretic, antifungal, and anti-inflammatory agents, applied in folk medicine as therapy for rheumatic fevers, diarrhea, malaria, hypertension, diabetes, and gastrointestinal diseases^2^. Many phytochemical studies on the isolation of compounds from the *Ajuga* genus have been conducted which resulted in the isolation and characterization of a series of secondary metabolites, including ecdysteroids, sesquiterpenoids, diterpenoids, triterpenoids, sterols, iridoids, withanolides, ionones and other compounds^3,4^.

*Ajuga turkestanica* (Regel) Briq. is naturally growing in Uzbekistan and Tajikistan^5,6^. *A. turkestanica* is an endemic Uzbek plant that contains ecdysteroids and iridoids. It is used in cosmetic products (creams, lotions etc.) to prevent signs of aging and as emollients, emulsifying and viscosity controlling agents^7–9^. The major bioactive constituents of *A. turkestanica* are represented by ecdysteroids and iridoids, and most of the studies described in the literature are focused on their identification and quantification. Researchers^6,10–16^ identified multiple ecdysteroids from *A. turkestanica*, among which turkesterone, 20-hydroxyecdysone, and cyasterone were the major compounds. Iridoid glucosides, such as harpagide and 8-*O*-acetylharpagide^17^ and several neo-clerodane diterpenoids: 14,15-dihydroajugachin B, 14-hydro-15-methoxyajugachin B, chamaepitin, ajugachin B, ajugapitin, and lupulin A, were isolated too^18,19^. *A. turkestanica* is used in folk medicine for its beneficial effects on muscle strength, muscular and stomach aches, and its protective action against heart diseases^18,19^. Several biological effects such as adaptogenic, anabolic, sedative, antifeedant and other activities have been documented concerning extracts and pure compounds isolated from this species^3^. Turkesterone and 20-hydroxyecdysone (ecdysterone) may also help promote lipid and carbohydrate metabolism, improve protein synthesis, aid muscle hypertrophy, and increase strength^20^. Many bodybuilding supplements containing these ecdysteroids can be found in "grey" online markets. Naturally, ecdysterone controls ecdysis (moulting) and metamorphosis of arthropods, but also caspase inhibition and autophagy induction are reported.

However, the other metabolites and quantity of major compounds present in different organs of *A. turkestanica*, as well as their effects against helminths, phytopathogens and cytotoxic properties as measured against selected cancer cells remain unknown. Such information is notably essential to get a deeper insight into the bioactive molecules in *A. turkestanica*. In this study, NMR spectroscopy and UHPLC-MS were used to characterize metabolites of different organs (flowers, fruits, leaves, roots, seeds, stems) of *A. turkestanica*. Moreover, the in vitro cytotoxic, antifungal, and anthelmintic properties of the methanolic extract obtained from this species were evaluated.

## Results and discussion

### UHPLC-MS experiments

LC-MS based metabolite profiling is the gold standard technique for characterization of specific metabolites in plants in a high-throughput manner. In this study, UHPLC-ESI-QqTOF-MS/MS was used as a tool for annotation and tentative identification of individual analytes. It allowed the identification of in total 51 metabolites in the flowers, fruits, leaves, roots, seeds, and stems of *A. turkestanica.* Their identification relied on exact *m/z* values obtained from MS spectra and fragmentation patterns derived from the results of MS/MS experiments, which were compared with the data available in the scientific literature and open-access databases/spectral libraries. The methanolic extracts obtained from the different organs of *A. turkestanica* were initially analyzed in both positive and negative ion modes. Compared to the positive ion mode, the MS spectra acquired in the negative ion mode were featured with higher intensity and higher number of detected signals.

**Fig. 1:**
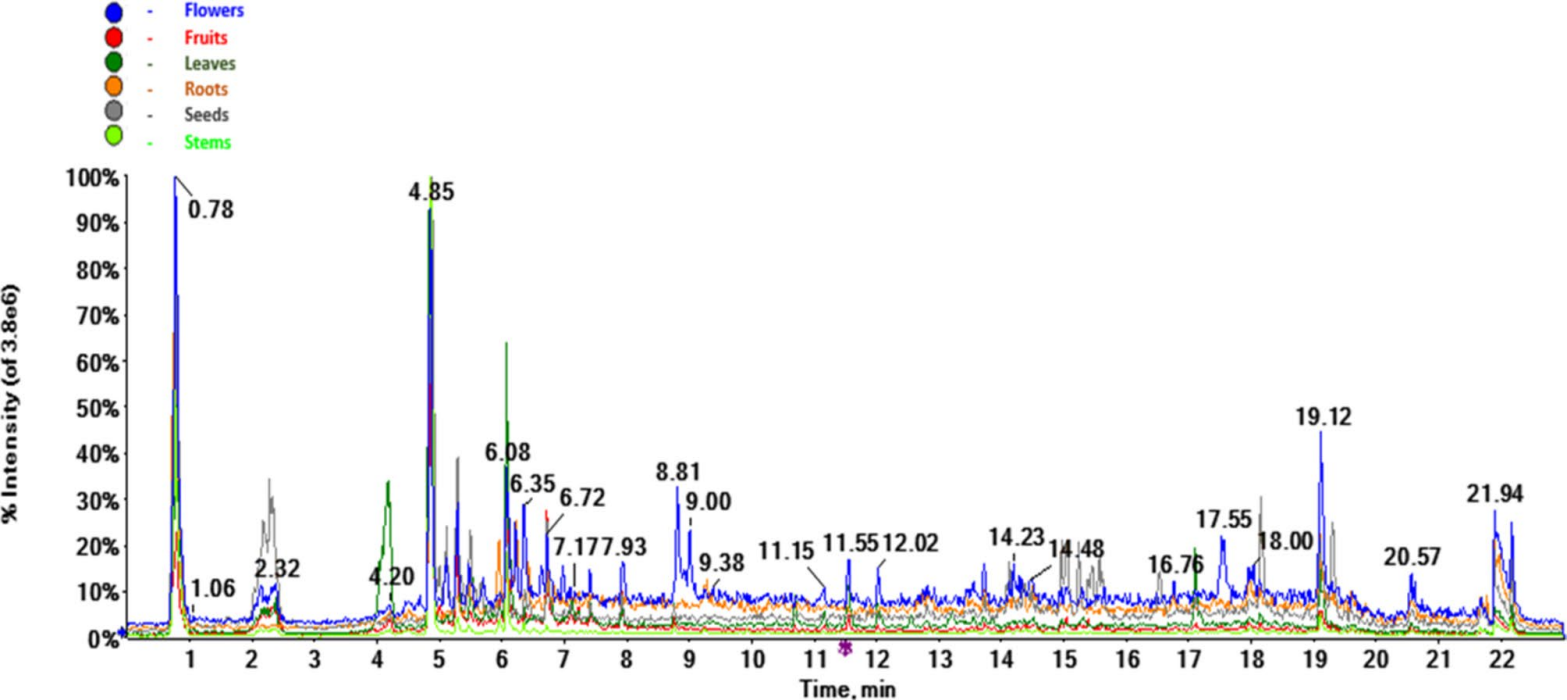
Representative RP-UHPLC-QqTOF-MS total ion chromatograms of methanolic extracts isolated from different organs of *A. turkestanica* acquired in negative ion mode.

Representative UHPLC-MS total ion chromatograms of *A. turkestanica* by organ type are presented in Fig. 1. Thereby, several ecdysteroids and iridoids could be identified by co-elution with authentic standards and comparison of their MS/MS spectra with those acquired with the standards. The retention times, elemental composition and *m/z* values of individual characteristic molecular ionic species are presented in Table 1. Metabolite assignments relied on the comparison of the experimental HR-QqTOF-MS and MS/MS spectra (accurate mass, isotopic distribution, and fragmentation patterns) with the literature data available for natural compounds detected in the *Ajuga* genus.

**Table 1:** Compounds identified in the methanolic extracts of *Ajuga turkestanica* (all organs).

Peak number/compound	R_t_, min	Compound identification (tentatively)	Molecular formula	Adduct ion	*m/z*	Accuracy (ppm)	MS/MS fragment ions	Reference *Ajuga* species (ref. cpd.)	Class of the compound
1	0.76	Tetrahexosaccharide	C_24_H_42_O_21_	[M + HCOO]^−^	711.2195	0.0			Sugars
2	0.77	D-Gluconic acid	C_6_H_12_O_7_	[M−H]^−^	195.0505	−1.0			Sugars
3	0.79	Sucrose	C_12_H_22_O_11_	[M−H]^−^	341.1084	0.3			Sugars
				[2M−H]^−^	683.2251	3.4			
4*	2.32	Harpagide	C_15_H_24_O_10_	[M + HCOO]^−^	409.1352	−2.8	363.1254, 219.1706	*A. turkestanica* ^17^	Iridoids
				[M + NH_4_]^+^	382.1708	0.9	329.1232, 185.0811, 167.0707		
5	4.14	*cis*-Melilotoside	C_15_H_18_O_8_	[M + H]^+^	327.1074	2.6	165.0569, 147.0456	*A. laxmannii* ^21,22^	Phenolics
				[M + NH_4_]^+^	344.1344	3.4			
6	4.50	Hexapyranosyl shanzhiside methyl ester	C_23_H_36_O_16_	[M−H]^−^	567.1931	0.1	457.1308		Iridoids
				[M + HCOO]^−^	613.1985	1.4			
7*	4.84	8-*O*-Acetylharpagide	C_17_H_26_O_11_	[M−H]^−^	405.1402	2.6	345.1124	*A. turkestanica* ^17^	Iridoids
				[2M−H]^−^	811.2877	3.3			
				[M + HCOO]^−^	451.1457	1.7			
				[2M + HCOO]^−^	857.2932	3.1			
				[M + NH_4_]^+^	424.1813	2.0	329.1237, 167.0705		
8	4.86	Melilotic acid	C_9_H_10_O_3_	[M + H]^+^	167.0708	0.3			Phenolics
9	4.88	Isomer of hexapyranosyl shanzhiside methyl ester	C_23_H_36_O_16_	[M−H]^−^	567.1931	0.4			Iridoids
				[M + HCOO]^−^	613.1989				
10	5.00	Turkesterone 22-acetate	C_29_H_46_O_9_	[M + HCOO]^−^	583.3118	0.3		*A. turkestanica* ^6^	Ecdysteroids
				[M−H]^−^	537.3063	2.3			
11	5.11	6-Deoxy-8-*O*-acetylharpagide	C_17_H_26_O_10_	[M−H]^−^	389.1453	−0.3	377.1765, 327.1072	*A. reptans* ^23,24^	Iridoids
				[M + HCOO]^−^	435.1508	−3.0			
				[M + NH_4_]^+^	408.1864	0.4	313.1278, 151.0752		
				[2M + NH_4_]^+^	798.3390	−1.2			
12*	5.28	Turkesterone	C_27_H_44_O_8_	[M + HCOO]^−^	541.3018	−3.0	325.0889	*A. turkestanica* ^6,11,12^	Ecdysteroids
				[2M + HCOO]^−^	1037.6054	−3.8			
				[M + H]^+^	497.3109	0.0	461.2897, 443.2789, 363.21.79		
				[M + NH_4_]^+^	514.3374	−1.1			
13	5.69	Kaempferol *O*-glucuronide	C_21_H_18_O_12_	[M + H]^+^	463.0877	1.0	287.0540		Flavonoids
14	5.70	Luteolin glucuronoside	C_28_H_14_O_7_	[M−H]^−^	461.0725	−3.6	406.0678, 285.0392, 230.0316		Flavonoids
15	5.78	25-Hydroxyatrotosterone A	C_28_H_46_O_8_	[M + HCOO]^−^	555.3175	0.4	367.1010, 329.0652	*A. turkestanica* ^6^	Ecdysteroids
16	5.95	Lavandulifolioside	C_34_H_44_O_19_	[M−H]^−^	755.2399	−2.3	565.1870, 523.2854, 431.0923, 377.1132	*A. turkestanica* ^25^	Phenylpropanoids
17*	6.08	20-Hydroxyecdysone	C_27_H_44_O_7_	[M + HCOO]^−^	525.3069	−3.2		*A. turkestanica* ^10^	Ecdysteroids
				[2M + HCOO]^−^	1005.6098	−5.2			
				[M + H]^+^	481.3183	3.7	445.2934, 449.1065, 427.2835, 347.2212		
				[2M + H]^+^	961.6215	−3.3			
18	6.20	Kaempferol 3-rutinoside-7-glucoside	C_33_H_40_O_20_	[M−H]^−^	755.2034	1.9	581.2877, 555.2720, 377.1112		Flavonoids
19*	6.23	Baicalin	C_21_H_18_O_11_	[M−H]^−^	445.0776	−3.5	269.0432, 222.0334		Flavonoids
20	6.35	Nepetin glucuronide	C_23_H_22_O_13_	[M−H]^−^	505.0988	−3.3	475.0831		Flavonoids
				[2M−H]^−^	1011.2048	1.5			
21	6.36	Methylscutellarin	C_22_H_20_O_12_	[M−H]^−^	475.0877	0.6	413.2144, 301.1266, 201.1116		Flavonoids
22	6.37	Acteoside (verbascoside)	C_29_H_36_O_15_	[M−H]^−^	623.1976	0.5	161.0280	*A. chamaepitys* ^26^	Phenylpropanoids
23	6.47	Leonoside A	C_35_H_46_O_19_	[M−H]^−^	769.2555	0.8		*A. turkestanica* ^25^	Phenylpropanoids
24	6.62	Kaempferol 3-rhamnoside	C_21_H_20_O_10_	[M−H]^−^	431.0984	−3.7	317.1937, 219.1744	*A. remota* ^27^	Flavonoids
25*	6.72	Cyasterone	C_29_H_44_O_8_	[M−H]^−^	519.2963	−3.6	501.2850	*A. turkestanica* ^10^	Ecdysteroids
				[M + H]^+^	521.3109	−1.1	503.3042, 485.2928, 467.2809, 303.1972		
				[2M + H]^+^	1041.6125	−1.9			
				[M + NH_4_]^+^	538.3374	−1.0			
26	7.11	Atrotosterone C	C_28_H_44_O_8_	[M−H]^−^	507.2963	−3.2	405.1740, 343.2097, 241.1063	*A. turkestanica* ^6^	Ecdysteroids
27	7.23	11-Hydroxy-Δ24-capitasterone	C_29_H_42_O_8_	[M−H]^−^	563.2862	−0.6	517.3008, 457.2420, 405.1762	*A. turkestanica* ^6^	Ecdysteroids
28	7.41	Ajugalactone	C_29_H_40_O_8_	[M−H]^−^	515.2650	−3.77	471.1268, 299.1832	*A. turkestanica* ^15^	Ecdysteroids
				[M + HCOO]^−^	561.2705	−3.4			
				[M + NH_4_]^+^	534.3061	−1.4	517.2788, 430.1698, 251.1850		
				[2M + NH_4_]^+^	1050.5785	−1.7			
29	7.85	Eriodictyol	C_15_H_12_O_6_	[M−H]^−^	287.0556	3.0	135.0448		Flavonoids
30*	7.92	Cyasterone 22-acetate	C_31_H_46_O_9_	[M−H]^−^	561.3069	−2.1	285.0383	*A. turkestanica* ^13^	Ecdysteroids
				[M + HCOO]^−^	607.3124	−2.6			
				[M + H]^+^	563.3215	−1.5	545.3085, 356.2635, 287.0544		
				[2M + H]^+^	1125.6294	−5.5			
				[M + NH_4_]^+^	580.3480	2.7			
31*	7.93	Luteolin	C_15_H_10_O_6_	[M + H]^+^	287.0556	3.2	153.0177	*A. chia* ^28^	Flavonoids
32*	8.81	Apigenin	C_15_H_10_O_5_	[M−H]^−^	269.0455	−2.6	117.0319	*A. chia* ^28^	Flavonoids
33	9.01	Hispidulin	C_16_H_12_O_6_	[M−H]^−^	299.0561	−2.7	269.0435, 209.9468		Flavonoids
				[2M−H]^−^	599.1195	−2.3			
34	9.13	14-Hydro-15-hydroxyajugapitin	C_29_H_44_O_11_	[M + NH_4_]^+^	586.3222	2.9	391.2113, 296.2070	*A. chamaepitys* ^29^	Diterpenoids
35	9.38	Chamaepitin	C_31_H_46_O_13_	[M + HCOO]^−^	671.2920	−2.1	553.2596, 299.0528, 269.0433, 213.1479	*A. chamaepitys* ^18^, *A. turkestanica* ^30^	Diterpenoids
36	10.27	14,15-Dihydroajugachin A	C_28_H_42_O_10_	[M + NH_4_]^+^	556.3116	2.5	539.2855, 391.2117, 291.1946, 158.1542	*A. bracteosa* ^31^, *A. reptans* ^32^	Diterpenoids
37	10.70	14,15-Dihydroajugachin B	C_31_H_46_O_12_	[M + HCOO]^−^	655.2971	2.5		*A. turkestanica* ^18^	Diterpenoids
				[2M + HCOO]^−^	1265.5961	2.2			
				[M + NH_4_]^+^	628.3328	2.3			
				[2M + NH_4_]^+^	1238.6317	2.6			
38	11.15	14,15-Dihydro-ajugapitin	C_29_H_44_O_10_	[M + HCOO]^−^	597.2917	−1.4	581.2588, 305.1727	*A. bracteosa* ^31^, *A. chamaepitys* ^31^, *A. pseudoiva* ^33^	Diterpenoids
				[M + NH_4_]^+^	570.3273	0.4	554.2971, 537.2695, 391.1217		
				[2M + NH_4_]^+^	1122.6207	−0.4			
39	11.30	14-Hydro-15-methoxyajugachin B	C_32_H_48_O_13_	[M + HCOO]^−^	685.3077	−2.7	653.2794, 619.3096, 409.1792, 341.1926, 309.2051	*A. turkestanica* ^18^	Diterpenoids
				[M + NH_4_]^+^	658.3433	−0.8	626.3175, 570.3279, 554.2969, 230.2486		
40	11.55	Ajugachin B	C_31_H_44_O_12_	[M + HCOO]^−^	653.2815	−2.3		*A. turkestanica* ^18^, *A. chamaepitys* var. *chia* ^34^, *A. forrestii* ^35^	Diterpenoids
				[M + NH_4_]^+^	626.3171	0.5			
				[2M + NH_4_]^+^	1234.6004	−1.2			
41	12.01	Ajugapitin	C_29_H_42_O_10_	[M + HCOO]^−^	595.2760	−3.2		*A. turkestanica* ^18^, *A. forrestii* ^35^	Diterpenoids
				[M + NH_4_]^+^	568.3116	0.0	551.2851, 389.1963		
				[2M + NH_4_]^+^	1118.5894	0.1			
42	12.55	Ajuganipponin B	C_29_H_40_O_9_	[M + HCOO]^−^	577.2654	−2.6	307.1849	*A. nipponensis* ^36^, *A. ciliata* ^37^	Diterpenoids
43	13.63	11-Hydroxycyasterone	C_29_H_44_O_9_	[M + NH_4_]^+^	554.3324	−2.6	538.3001, 357.1465, 330.2096, 258.2790	*A. turkestanica* ^6^	Ecdysteroids
44	13.71	4-Methylumbelliferyl-β-D-glucopyranoside	C_16_H_18_O_8_	[M + H]^+^	339.1080	1.8			Coumarins
45	14.12	12,13-Epoxy-9(*Z*)-octadecenoic acid	C_18_H_32_O_3_	[M−H]^−^	295.2273	0.3	277.2502		Fatty acids
46	14.48	Myristyl sulfate	C_14_H_30_O_4_S	[M−H]^−^	293.1787	2.2			Fatty acids
47	15.42	10-Methylundecanoic acid	C_12_H_24_O_2_	[M−H]^−^	199.1698	0.7			Fatty acids
48	17.55	Ursolic acid	C_30_H_48_O_3_	[M−H]^−^	455.3531	−2.3	325.1797, 255.2325	*A. chamaepitys* ssp. *laevigata* ^38^	Triterpenes
49	18.16	Linoleic acid	C_18_H_32_O_2_	[M−H]^−^	279.2324	2.0			Fatty acids
50	18.30	Pentadecanoic acid	C_15_H_30_O_2_	[M−H]^−^	241.2168	1.8			Fatty acids
51	19.30	Stearic acid	C_18_H_36_O_2_	[M−H]^−^	283.2640	1.1			Fatty acids

^*^Annotation of these compounds was confirmed by coelution with authentic standards.

**Fig. 2:**
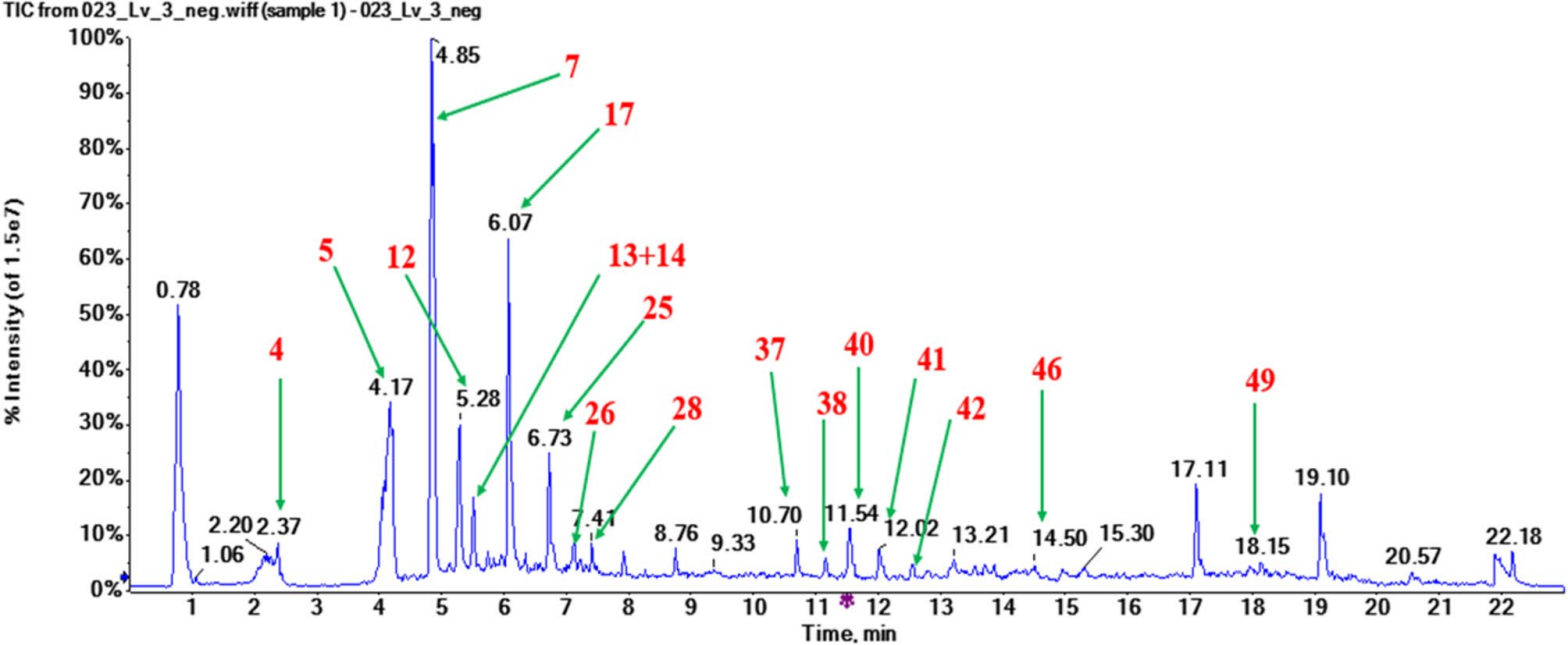
RP-UPLC-QqTOF-MS total ion chromatogram of the methanolic extract obtained from the leaves of *A. turkestanica* acquired in MS full scan (TOF-scan) mode in the *m/z* range 50–1250. Several peaks have been annotated in the chromatogram with the names of the metabolites shown in Table 1.

**Fig. 3:**
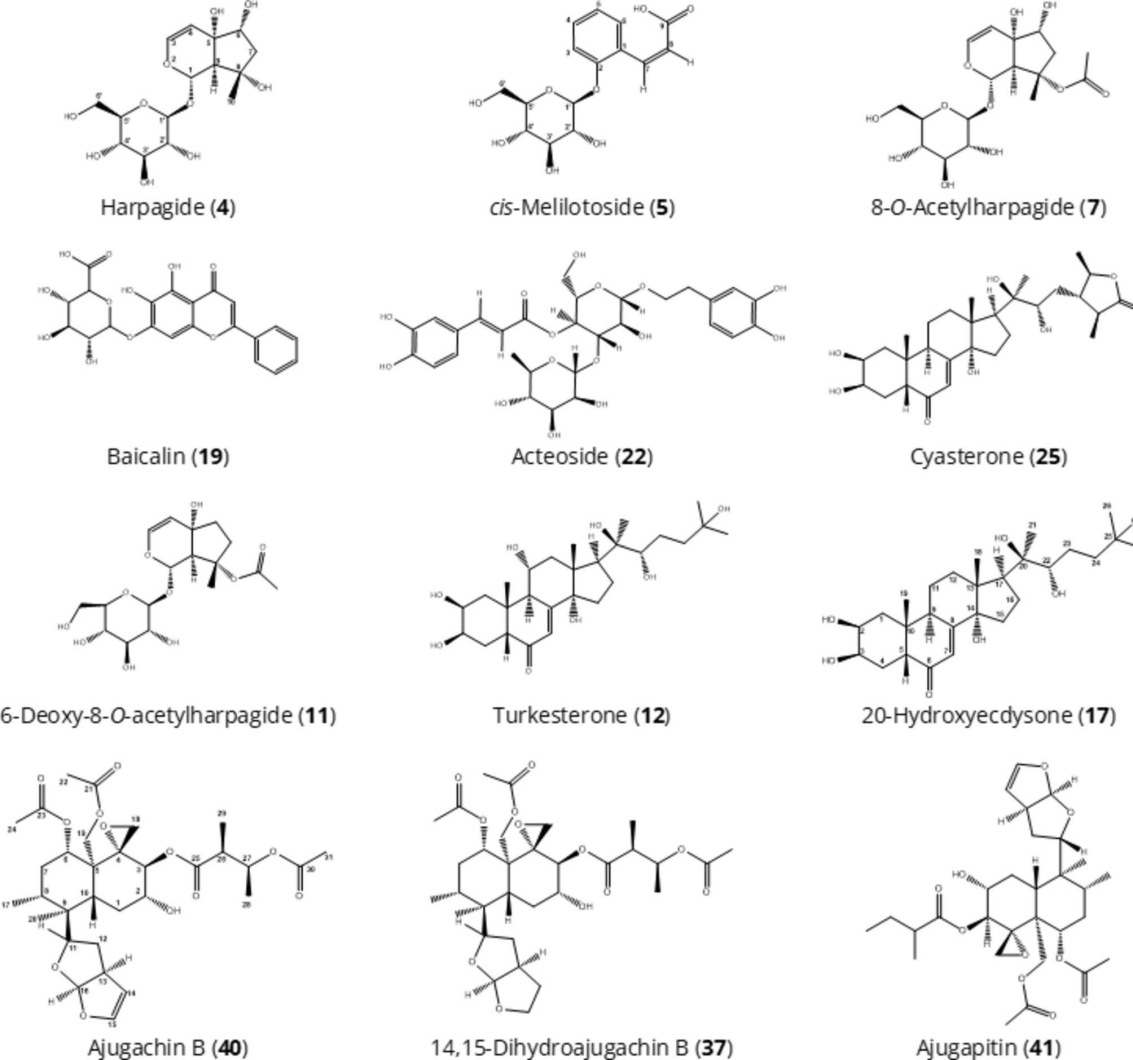
Chemical structures of the major compounds of the *A. turkestanica* extracts.

A total of 51 compounds belonging to various phytochemical classes (11 flavonoids, 10 ecdysteroids, 9 diterpenes, 6 fatty acids, 5 iridoids, 3 phenylpropanoids, 3 sugars, 2 phenolics, 1 coumarin, 1 triterpene) could be annotated and tentatively identified in the methanolic extracts obtained from the organs of *A. turkestanica* using the LC-ESI-QqTOF-MS and MS/MS (Table 1, Fig. 2). Chemical structures of the identified major phytoconstituents are shown in Fig. 3.

### Identification of the ecdysteroids

In this study, altogether ten ecdysteroids were identified by UHPLC-ESI-MS/MS in *A. turkestanica* for the first time (Table 1). These ecdysteroids, namely 20-hydroxyecdysone (peak number **17**), cyasterone (**25**)^32^, turkesterone (**12**)^11,12^, ajugalactone (**28**)^15^, cyasterone 22-acetate (**30**)^13^, turkesterone 22-acetate (**10**), 11-hydroxy-sidisterone, 22-oxo-turkesterone, atrotosterone C (**26**), 11-hydroxy-Δ24-capitasterone (**27**), 25-hydroxyatrotosterone A, 11-hydroxycyasterone (**43**), abutasterone, 25-hydroxydacryhainansterone, ajugasterone C^6^ had previously been isolated and characterized from *A. turkestanica* by other methods than UHPLC-ESI-MS/MS. Here these compounds were confirmed with the LC-MS approach. 20-Hydroxyecdysone (**17**, C_27_H_44_O_7_) was the second major compound, detected in all plant organs besides roots, and was represented in the mass spectra by the signals at *m/z* 525.3069 and *m/z* 1005.6098 corresponding to the formate [M + HCOO]^−^ and [2M + HCOO]^−^. In the positive ion mode, the [M + H]^+^at *m/z* 481.3183 demonstrated a fragmentation pattern typical for 20-hydroxyecdysone (Fig. S4b). The spectrum is dominated by a signal at *m/z* 961 (corresponding to [2M + H]^+^) and a characteristic pattern of neutral losses at *m/z* 943 ([2M + H–H_2_O]^+^), 925 ([2M + H−2H_2_O]^+^), 463 ([MH−H_2_O]^+^), *m/z* 445 ([MH−2H_2_O]^+^), and *m/z* 427 ([MH−3H_2_O]^+^) was observed. Based on the comparison of stacked experimental and reference mass spectra, this peak was annotated as belonging to 20-hydroxyecdysone (**17**). The peak **25** corresponded to [M−H]^−^ at *m/z* 519.2963, [M + H]^+^ at *m/z* 521.3109, [M + NH_4_]^+^ at *m/z* 538.3374, and based on their fragmentation was identified as cyasterone (**25**). In the total ion chromatogram (TIC) of the seed methanolic extract, cyasterone represented the major peak. Similarly, turkesterone (**12**, C_27_H_44_O_8_) was identified as peak **12** based on the signals at *m/z* 541.3018 corresponding to its [M + HCOO]^−^ and at *m/z* 1037.6054 corresponding to [2M + HCOO]^−^ in negative ion mode. In positive ion mode turkesterone (**12**) was observable as a pattern of ionic adducts–[M + H]^+^ at *m/z* 497.3109 and [M + NH_4_]^+^ at *m/z* 514.3374. Moreover, this pattern was accompanied by several water loss signals at *m/z* 479 ([MH−H_2_O]^+^), 461 ([MH−2H_2_O]^+^), and 443 ([MH−3H_2_O]^+^). The presence of turkesterone (**12**) was confirmed by comparison with the reference spectra (Fig. S3a–b).

### Identification of the diterpenes

Based on the combination of the fragmentation patterns acquired by UHPLC-MS/MS and available bibliographic data, 9 diterpenes could be annotated in *A. turkestanica*. Thus, chamaepitin (**35**), 14,15-dihydroajugachin B (**37**), 14-hydro-15-methoxyajugachin B (**39**), ajugachin B (**40**), ajugapitin (**41**) have already been isolated from *A. turkestanica*^18^. On the other hand, 14-hydro-15-hydroxyajugapitin (**34**)^29^, 14,15-dihydroajugachin A (ajubractin D) (**36**)^31,32^, 14,15-dihydro-ajugapitin (**38**)^31,33^, ajuganipponin B (**42**)^34,37^ were previously found in other *Ajuga* species.

The UHPLC-MS analysis of the compound **40** gave [M + HCOO]^−^ at *m/z* 653.2815, [M + NH_4_]^+^ at *m/z* 626.3171, [2M + NH_4_]^+^ at *m/z* 1234.6004 and was annotated as ajugachin B (C_31_H_44_O_12_) by comparison with the literature data^18^. Compound **37** (C_31_H_46_O_12_) could be observed in the MS spectra as the pattern of [M + HCOO]^−^ at *m/z* 655.2971, [2M + HCOO]^−^ at *m/z* 1265.5961, [M + NH_4_]^+^ at *m/z* 628.3328 and [2M + NH_4_]^+^ at *m/z* 1238.6317. Based on the comparison with the literature data^18^, the compound was tentatively assigned as 14,15-dihydroajugachin B. The peak **41** yielded a pattern of signals at *m/z* 595.2760 ([M + HCOO]^−^), *m/z* 568.3116 ([M + NH_4_]^+^) and *m/z* 1118.5894 ([2M + NH_4_]^+^) observable in the negative and positive ion mode. With consideration of the litarature data^18^ this compound was annotated as ajugapitin (C_29_H_42_O_10_). Ajugachin B (**40**), ajugapitin (**41**) and 14,15-dihydroajugachin B (**37**) were observed as the major compounds in the flowers and leaves of *A. turkestanica*.

### Identification of the iridoid glycosides

Regarding iridoids, harpagide (**4**, C_15_H_24_O_10_, *m/z* 409.1352 [M + HCOO]^−^) and 8-*O*-acetylharpagide (**7**, C_17_H_26_O_11_, *m/z* 382.1708 [M + NH_4_]^+^) were previously isolated from *A. turkestanica* by Kotenko et al.^17^ 6-Deoxy-8-*O*-acetylharpagide (**11**, C_17_H_26_O_11_, *m/z* 405.1402 [M−H]^−^, *m/z* 424.1813 [M + NH_4_]^+^) was previously isolated from *A. reptans*^23,24^. In this study, hexapyranosyl shanzhiside methyl ester and its isomer (**6** and **9,** respectively, C_23_H_36_O_16_, *m/z* 567.1931 [M−H]^−^) were annotated here in *A. turkestanica* for the first time. Our relative quantification data showed that 8-*O*-acetylharpagide (**7**) was the most abundant constituent in the methanolic extracts of the leaves followed by flowers. The major iridoid 8-*O*-acetylharpagide (**7**) was identified by the characteristic patterns of negatively charged ionic species: [M−H]^−^ (*m/z* 405.1402), [M + HCOO]^−^ (*m/z* 451.1457), [2M−H]^−^ (*m/z* 811.2877), [2M + HCOO]^−^ (*m/z* 857.2932) and [M + NH_4_]^+^ (*m/z* 424.1813) (see Fig. S1 for the spectrum of **7** with negative ionization). The highest contents of harpagide (**4**) were detected in the methanolic extracts of the seeds and flowers (Table 1 and Figs. 1, 2).

### Identification of the phenylethanoids, flavonoids and phenolic acids

Several subclasses of annotated polyphenols (including flavonoids, phenylethanoid glycosides, and phenolic acids) are summarized in Table 1. Thereby, flavonoids, phenylethanoids, and phenolic acids (represented with eleven, three and two species, respectively) were the best represented classes. The MS/MS analyses led to a successful identification of the aglycone moieties. Based on the presence of characteristic neutral losses of 176, 162, 146, and 132 u clearly visible in the MS/MS spectra, the sugar part in the *O*-glycosides could be annotated as hexuronic acid, hexose, deoxyhexose, and pentose, respectively. Interpretation of the MS spectra led to the identification of three kaempferol conjugates (aglycon signal at *m/z* 285 in the MS/MS spectra) including kaempferol glucuronoside (**13**, C_21_H_18_O_12_, [M + H]^+^ at *m/z* 463.0877), kaempferol 3-rutinoside-7-glucoside (**18**, C_33_H_40_O_20_, [M−H]^−^ at *m/z* 755.2034), and kaempferol 3-rhamnoside (**24**, C_21_H_20_O_10_, [M−H]^−^ at *m/z* 431.0984). Kaempferol 3-rhamnoside (**24**, C_21_H_20_O_10_, [M−H]^−^ at *m/z* 431.0984) was isolated from *A. remota*^27^. The phenylethanoid glycosides lavandulifolioside (**16**, C_34_H_44_O_19_, [M−H]^−^ at *m/z* 755.2399) and leonoside A (**23**, C_35_H_46_O_19_, [M−H]^−^ at *m/z* 769.2555) were isolated from *A. turkestanica*^25^, while acteoside (verbascoside) (**22**, C_29_H_36_O_15_, [M−H]^−^ at *m/z* 623.1976) was observed in *A. chamaepitys*^26^. The highest contents of acteoside (**22**) were observed in the roots of *A. turkestanica*. On the other hand, *cis*-melilotoside (**5**, C_15_H_18_O_8_, [M + H]^+^ at *m/z* 327.1074) was observed as the major compound only in the leaves of *A. turkestanica*. Free aglycones were luteolin (**31**, C_15_H_10_O_6_, [M + H]^+^ at *m/z* 287.0556), apigenin (**32**, C_15_H_10_O_5_, [M−H]^−^ at *m/z* 269.0455), and eriodictyol (**29**, C_15_H_12_O_6_, [M−H]^−^ at *m/z* 287.0556) in the flower extract, in addition to hispidulin (**33**, C_16_H_12_O_6_, [M−H]^−^ at *m/z* 299.0561), baicalin (**19**, C_21_H_18_O_11_, [M−H]^−^ at *m/z* 445.0776), and nepetin 7-glucuronide (**20**, C_23_H_22_O_13_, [M−H]^−^ at *m/z* 505.0988). Baicalin (**19**) was found as a major compound in the roots of *A. turkestanica*. Several compounds, namely melilotic acid (**8**), kaempferol glucuronoside (**13**), luteolin-glucuronoside (**14**), kaempferol 3-rutinoside-7-glucoside (**18**), baicalin (**19**), nepetin glucuronide (**20**), methylscutellarin (**21**), eriodictyol (**29**), hispidulin (**33**) are reported in *Ajuga* here for the first time. The overall phenylpropanoid relative contents in the organs of *A. turkestanica* can be ranked as flowers > leaves > roots > fruits > seeds and stems (Figs. 1, 2).

### Identification of the fatty acids

Six fatty acids and their derivatives were characterized in *A. turkestanica*. Lipid composition of the aerial parts, flowers, roots, and seeds of this plant had been described by Khidoyatova et al.^39^ earlier. More than half of the total fatty acid contents in *A. turkestanica* aerial parts is represented by unsaturated acids, including oleic and linolenic acid. The fatty acids isolated from flowers were enriched in saturated components, with palmitic acid dominating^39^. Our studies showed that the amounts of fatty acids expectedly were significantly higher in seeds than in other parts (Fig. 1). In this study, on the basis of fragmentation patterns and molecular compositions, the fatty acids were putatively identified as 12,13-epoxy-9-octadecenoic acid (**45**, [M−H]^−^ at *m/z* 295.2273), myristyl sulfate (**46**, [M−H]^−^ at *m/z* 293.1787), 10-methylundecanoic acid (**47**, [M−H]^−^ at *m/z* 199.1698), linoleic acid (**49**, [M−H]^−^ at *m/z* 279.2324), pentadecanoic acid (**50**, [M−H]^−^ at *m/z* 241.2168) and stearic acid (**51**, [M−H]^−^ *m/z* 281.2481) (Table 1).

### NMR investigation

**Fig. 4:**
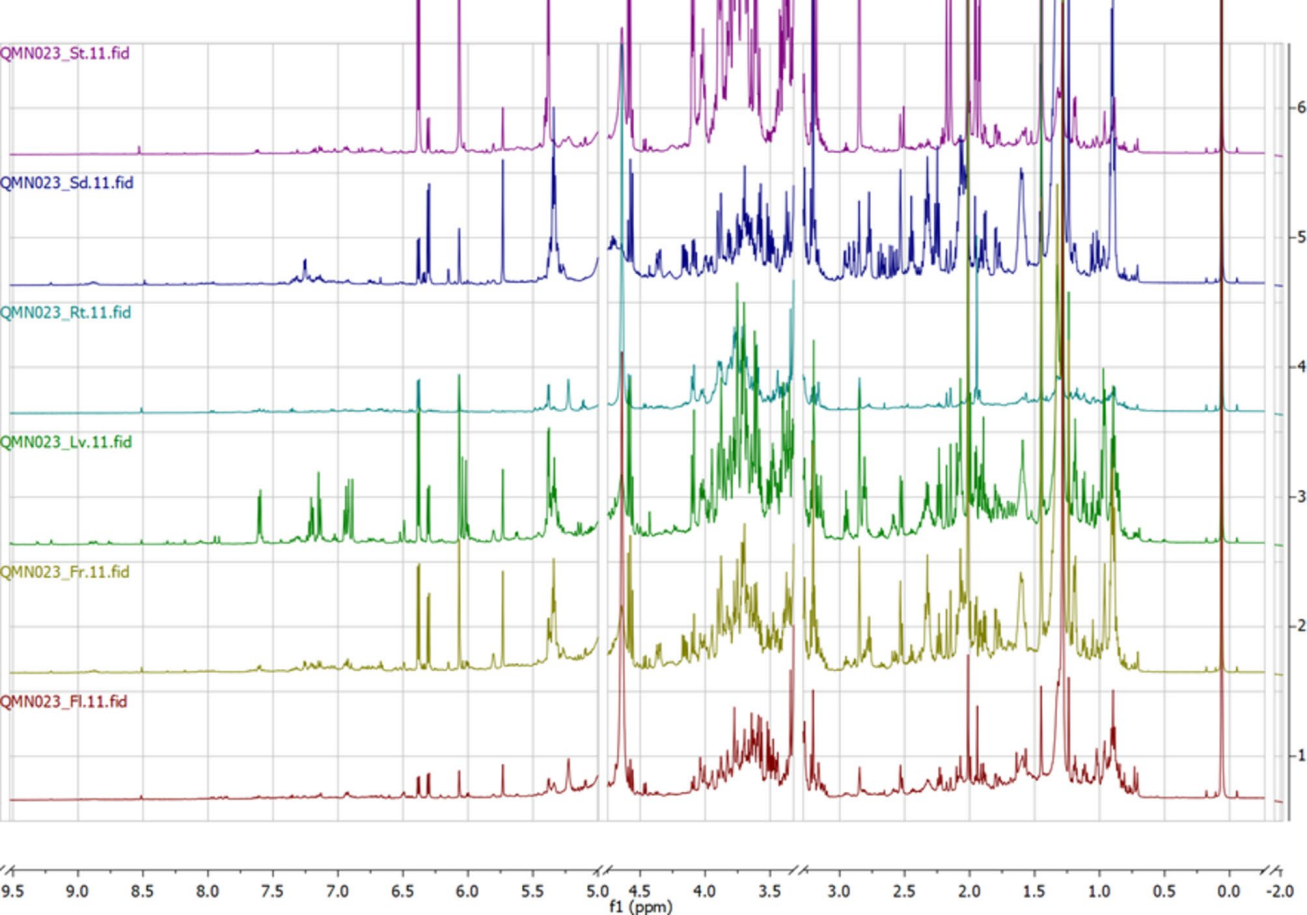
Stacked ^1^H NMR spectra of the methanol extracts obtained from different organs of *A. turkestanica* showing from top to bottom extract from: stems (violet), seeds (blue), roots (turquoise), leaves (green), fruits (olive drab), and flowers (maroon). The leaves extract (green) showed a higher content of secondary metabolites.

Expectedly, the ^1^H NMR spectra of the crude methanol extracts were composed of crowded aliphatic and aromatic spectral regions (the stacked ^1^H spectra are depicted in Fig. 4). The 2D NMR spectra of leaves and stems and the comparison of experimental and literature data allowed an identification of harpagide (**4**), 8-*O*-acetylharpagide (**7**), sucrose (**3**), *cis*-melilotoside (**5**), ajugachin B (**40**) and 20-hydroxyecdysone (**17**). The initial step in the identification process of harpagide (**4**) and 8-*O*-acetylharpagide (**7**) was the observation of 3 reliable HMBC correlations from their singlet-shaped CH_3_-groups resonating at *δ* = 1.24 and 1.45 ppm, respectively. According to HSQC, these three interacting carbons were a CH_2_-, CH-, and a non-protonated carbon bearing an oxygen atom,— the latter based on its ^13^C chemical shift (78.1 ppm for harpagide (**4**), and 88.6 ppm for 8-*O*-acetylharpagide (**7**)). The combined analysis of the COSY, HSQC, HMBC and TOCSY spectra proved the presence of the compounds’ iridoid core. The difference between the chemical shifts of C-8 of the two compounds indicated a possible substitution of the hydroxyl-group in position 8. Indeed, thanks to the relatively high concentration of 8-*O*-acetylharpagide (**7**), a minor ^4^*J*_CH_ correlation was visible between its acetyl protons and C-8. The observation of a pair of HMBC correlations between C-1 and H-1’, and C-1’ and H-1 allowed the identification of the linkage of the sugar unit through its anomeric position onto the iridoid aglycon. From the ^3^*J*_HH_ coupling constant values typical for proton pairs in axial positions of a pyranose chair, it was assumed that the monosaccharide unit could be a β-D-glucopyranose. However, this assumption could not be explicitly confirmed due to the strong coupling regime between H-4’ and H-5’ (both resonating at *δ*_*H*_ = 3.29 ppm in case of 8-*O*-acetylharpagide (**7**)), but the hypothesis was later proven by the comparison of experimental and literature NMR data^40^.

The identification process of *cis*-melilotoside (**5**) was initiated by the signal assignment of its *o*-disubstituted benzene ring. The HMBC correlations observed between two olefinic protons (H-7: *δ*_*H*_ = 6.03 and H-8: 6.90 ppm; both doublets with ^3^*J*_HH_ = 12.7 Hz) and carbons from the *o*-disubstituted benzene ring (C-1: *δ*_*C*_ = 127.8ppm for the former proton; C-6: *δ*_*C*_ = 131.0 and C-2: 156.2 ppm for the latter proton), and a carbonyl group (C-9: *δ*_*C*_ = 175.2 ppm) provided the assumption that the aromatic fragment is a substituted *o*-coumaroyl moiety. It was possible to unambiguously elucidate the substitution by observing an HMBC correlation between the proton H-1’ with *δ*_*H*_ = 4.93 ppm and the carbon *δ*_*C*_ = 156.2 ppm, together with the subsequent analysis of other homonuclear and heteronuclear correlations. A monosaccharide unit was suggested to be present within the molecule, specifically β-D-glucopyranose as implied by large *J*_HH_ values. A fair agreement of experimental and literature data could be found^41^. The coumaric acid derivative *cis*-melilotoside was isolated previously from *A. laxmannii* and *A. chamaecistus* ssp. *tomentella*^22,42^.

The high number of CH_3_-groups and the HMBC correlations with their equivalent protons, and especially the reliability of the information obtained therefrom, were key to the identification process of ajugachin B (**40**) and 20-hydroxyecdysone (**17**). Most structural fragments within the molecule were identified and successfully interconnected. However, the explicit determination of the two structures just from the acquired experimental NMR data was not possible. For 20-hydroxyecdysone (**17**) this was caused by the signal overlap in the aliphatic spectral region, whereas for ajugachin B (**40**) the uncommon epoxide ring caused troubles in the identification process. The presence of the latter stretched cycle could be easily proven by the extraction of its CH_2_-group’s ^1^*J*_CH_ interaction constant value from f2-coupled HSQC spectrum, but such a spectrum was not obtained during the NMR data acquisition. It was still possible to suggest structures limited by the structural restraints obtained from 2D homonuclear and heteronuclear NMR correlations and to obtain a good agreement of experimental NMR data with the literature data for ajugachin B (**40**)^34^ and 20-hydroxyecdysone (**17**)^43^.

Naturally, a plethora of signals of other metabolites was present in the ^1^H NMR spectra, but unfortunately the structures could not be unambiguously confirmed due to signal overlaps and similarities between compounds of varied chain lengths, configurational or constitutional isometry, and minor differences in substitution. Signals possibly arising from various phenolic compounds, and other ecdysteroids were observed, but it was not possible to safely determine their identities in the complex mixture. Unsaturated alkene chains were observed in some plant parts, possibly arising from the presence of unsaturated fatty acids, but it was impossible to identify the specific compounds. The presence of GABA was also suggested from the ^1^H and ^13^C signals and HMBC connectivity, but due to the relatively weak integral intensity it was not possible to safely determine, that the amino- and carboxylic acid moiety were unsubstituted. Despite GABA being a very common compound found in living organisms, its NMR data in the literature are mostly obtained from an aqueous solution or mixture. The presence of sucrose in the extracts of *A. turkestanica* was expected and was confirmed by the comparison of the NMR spectra of the extracts and the spectrum of a sucrose reference (**3**) dissolved in MeOH-*d*_4_.

It is well known that quantitative analysis by mass spectrometric methods is rather difficult, especially without the possibility to apply the method of standard addition. As discussed in previous sections, selected compounds were identified as the major constituents of the individual extracts, e.g. by LC. For this most relevant group it was decided to apply NMR for a reliable quantification. The ^1^H NMR spectra were measured with a 90-degree pulse and a 30-second-long repetition time to have the T1-relaxation process finished at the beginning of each scan. Up to 3 resolved signals were chosen for each identified compound (Table S7). Any potential overlap with another major compound was checked using the HSQC spectrum in the case of the extract from the leaves and stems of *A. turkestanica*. The approximate concentrations of the identified compounds are summarized in Table S7. Despite the quantitative values being only approximate, for several compounds the results show a discrepancy to values suggested by mass spectrometry. 8-*O*-Acetylharpagide (**7**) was found to dominate within the composition of the methanolic extracts of leaves, stems, and fruits. Harpagide (**4**) was found to be the major compound in the extract from the seeds of *A. turkestanica*. The flower and root extracts visibly showed a lower content of secondary metabolites both at first glance and after the quantification process. The presence of 20-hydroxyecdysone (**17**) could only be confirmed in the extracts from stems, leaves, and fruits—the signals thereof were either below the limit of detection or were overlapped in the other extracts. It should be noted that the values in Table S7 correspond to extracts prepared as described in the Materials and Methods section—the obtained concentrations are limited by the solubility of the individual components in 1 mL of MeOH-*d*_4_. The apparent simplicity of the quantification of the major components is an excellent basis for further work to create a robust, reproducible and validated analytical method.

### Biological evaluations

The methanolic extract of *A. turkestanica* was evaluated for its antifungal activity against the phytopathogens *Septoria triciti*, *Botrytis cinerea*, and *Phytophthora infestans* (Fig. S17). The results show that methanolic extract at the concentrations of 42 and 125 μg/mL did not inhibit *S. triciti* and *P. infestans*. On the other hand, the methanolic extract caused a 60% growth inhibition of *B. cinerea* culture at 125 µg/mL. The results of the cytotoxic assays showed that the methanolic extracts did not show any cytotoxic activity against PC-3 (prostate cancer) and HT-29 (colon adenocarcinoma) cells at a concentration of 0.05 µg/mL (Fig. S18). On the other hand, well-pronounced cytotoxic effects were observed for the extracts at the very high concentration of 50 µg/mL in MTT and CV assays. The anthelmintic activity of the methanolic extracts prepared from different organs of *A. turkestanica* were tested against *Caenorhabditis elegans* (Fig. S19). *C. elegans* is a non-pathogenic model test organism which can be used for the initial screening of anthelmintic compounds. The results of anthelmintic assays showed that the samples did not significantly kill *C. elegans* even at the highest concentration of 500 µg/mL. The results of bioassays corroborate the non-toxicity of *A. turkestanica* for human consumption, e.g., as used in (biologically active) supplements.

## Conclusion

This is the first profiling study of the secondary metabolites of the different organs of *A. turkestanica*. A total of 51 compounds were tentatively annotated by their accurate masses and tandem fragmentation patterns acquired by UHPLC-MS/MS experiments and some of them were identified by co-elution with authentic standards. Expectedly, the contents of the identified compounds varied in flowers, fruits, leaves, roots, seeds, and stems of *A. turkestanica*. NMR of crude extracts was found to be suitable for the quantification of the major metabolites. The results show that 8-*O*-acetylharpagide, 20-hydroxyecdysone and ajugachin B are the most abundant constituents in the species. The two major compounds 8-*O*-acetylharpagide and 20-hydroxyecdysone were chosen as quality markers for *A. turkestanica* preparations.

## Materials and methods

### Chemicals

Methanol, acetonitrile (both LC-MS CHROMASOLV™) were purchased from Riedel-de Haёn/Honeywell (Seelze, Germany), ultrapure water (resistivity ≥ 18 mΩ/cm) was purified in-house with GenPure Pro UV-TOC system (Thermo Scientific, Langenselbold, Germany), ammonium formate (≥ 99%, MS eluent additive) was obtained from Sigma Aldrich (Steinheim, Germany), formic acid (LC-MS grade, 98–100%) from Fluka™/Honeywell (Seelze, Germany). Epoxiconazole, terbinafine and ivermectin were purchased from Merck KGaA (Darmstadt, Germany). Deuterated methanol (MeOH–*d*_4_) and the internal standard hexamethyldisiloxane (HMDS) were obtained from Deutero (Germany). Reference compounds (turkesterone, 20-hydroxyecdysone, cyasterone, cyasterone-22-acetate, harpagide, 8-*O*-acetylharpagide, baicalin, luteolin, apigenin) obtained from the Institute of the Chemistry of Plant Substances (Tashkent, Uzbekistan) were used in this study with purities > 98%.

### Plant material

To obtain the plant material, aerial parts and roots of *A. turkestanica* were collected in the Baysun district of Surxondaryo region, Uzbekistan, during the flowering period on May 20th, 2019. The plant was identified by Dr. O.A. Nigmatullaev, Department of Herbal Plants (Institute of the Chemistry of Plant Substances, Uzbekistan) and a voucher specimen (number 2019064) was deposited at the Herbarium of this department. Sampling was conducted for academic purposes, with the permission of the Institute and in accordance with relevant institutional, national, and international guidelines and legislation, including the IUCN guidelines on research on endangered species and the Convention on the Trade in Endangered Species of Wild Fauna and Flora. The collected plant materials were washed with water, then cut into small pieces, and dried in the shade at room temperature for 2 weeks. Until use, the dried plant material is stored in sealed polyethylene bags with silica gel at −20 °C.

### Sample preparation for UHPLC-MS analysis

The dried and powdered aerial parts and roots of *A. turkestanica* (4 mg) were extracted with 2 mL of LC-MS grade methanol using an ultrasonic bath for 10 min at room temperature, and then centrifuged at 14000 g for 10 min to remove debris. After centrifugation, the supernatants were transferred to HPLC vials. This solution (concentration 2 mg/mL) was used for the QqTOF-MS measurements.

### ESI-QqTOF-MS and MS/MS measurements

5 µL of the methanolic extract of *A. turkestanica* were injected (partial loop injection mode) into a Waters Acquity UPLC I class system and separated on Nucleoshell RP18 column (150 mm × 2 mm, particle size 2.7 µm; Macherey-Nagel, Düren, Germany) at 40 °C. Eluents A and B were aqueous 0.3 mmol/L ammonium formate and acetonitrile, respectively. After a 2 min isocratic period at 5% eluent B, elution was accomplished at 0.4 mL/min in a linear gradient to 95% B within 17 min. After a 2 min column wash with 95% eluent B, the eluent composition was re-set to 5% eluent B and the column was re-equilibrated for 9 min. The autosampler temperature was set to 4 °C. The chromatographic effluents were on-line infused in a hybrid quadrupole-time of flight (QqTOF) mass spectrometer (AB Sciex TripleTOF 6600 system, AB Sciex, Darmstadt, Germany) equipped with a DuoSpray electrospray ionization (ESI) ion source and operated in sequential window acquisition of all theoretical fragment-ion spectra (SWATH) mode. All analyses were performed at the high sensitivity mode for both TOF and product ion scans. The mass calibration was automatically performed every 10 injections and relied on the Sciex APCI calibrant solution delivered via a calibration delivery system (CDS). The MS parameters were set as follows: MS^1^ accumulation time, 150 ms; MS^2^ accumulation time, 20 ms; collision energy, −45 V; collision energy spread, 35 V; cycle time, 1160 ms; Q1 window, 25 Da; mass range, *m/z* 65-1250. The other parameters were set as the following: curtain gas, 35 psig; ion source gas 1 (nebulizer), 60 psig; ion source gas 2 (drying gas), 70 psig; source temperature, 600 °C; ion spray voltage, −4.5 kV; declustering potential, 35 V. Kinetin (20 pmol per injection) was used as an internal standard. For interpretation of the LC-MS data PeakViewTM (version 2.2) tool (AB Sciex, Darmstadt, Germany) was used. All UHPLC-MS raw data, acquisition parameters and processing parameters are available on request from the corresponding author.

### Preparation of samples for NMR analysis

50 mg of aerial parts of *A. turkestanica* were extracted with 1 mL MeOH-*d*_4_ (99.8%) containing 0.93 mmol/L hexamethyl disiloxane (HMDS) in an ultrasonic bath for 10 min and then centrifuged for 15 min. After centrifugation, the supernatants were placed in NMR tubes and were left to stand overnight at 24 ℃.

### NMR measurements and processing

^1^H NMR spectra of the crude methanol extracts were measured at 25 °C on a Bruker Avance Neo NMR instrument (500 MHz) (Bruker, Karlsruhe, Germany) using 160 scans, 26.7 s relaxation delay, 3.3 s acquisition time, and a 90° pulse angle. The spectra were referenced to HMDS at 0.062 ppm for ^1^H NMR and 1.96 ppm for ^13^C NMR. The ^1^H NMR spectra have been phase and baseline corrected. Additionally, 2D NMR (HSQC, HMBC, COSY, TOCSY) spectra were obtained from the crude methanol extracts of leaves and stems. All NMR raw data, acquisition parameters and processing parameters are available on request from the corresponding author. The ^1^H NMR spectra were integrated by the line fitting algorithm included in the MNova 12 software package (Mestrelab Research, S.L., Santiago de Compostela, Spain).

### Assays for cytotoxic activity

The human prostate cancer cell line (PC3) and human colon adenocarcinoma cancer cell line (HT-29) were obtained from American Type Culture Collection (ATCC) and cultured under recommended conditions. The cytotoxicity of the methanolic extract at the concentrations of 0.05 and 50 µg/mL was evaluated against the PC-3 and HT-29 cell lines. The cell maintenance and assay procedures were performed as described by dos Santos et al.^44^. The viability of the cells was determined by MTT (3-(4,5-dimethylthiazol-2-yl)-2,5-diphenyl-2H-tetrazolium bromide) and CV (crystal violet) assays after 72 h incubation time. The absorbance was measured with an automated microplate reader at 540 nm with a reference wavelength of 670 nm. The results are presented as a percentage of control values obtained from untreated cultures.

### Assays for antifungal activity

The methanol extract was tested in 96-well microtiter plate assays against the phytopathogenic ascomycetes *Botrytis cinerea* Pers. and *Septoria tritici* Desm. and the oomycete *Phytophthora infestans* (Mont.) de Bary according to the monitoring methods approved by the fungicide resistance action committee (FRAC) with minor modifications as described before^45^. The crude extract was examined at a final concentration of 41.7 and 125 µg/mL. The solvent DMSO was used as negative control (max. concentration 2.5%), and the commercially used fungicides epoxiconazole and terbinafine (Sigma-Aldrich, Darmstadt, Germany) served as positive controls. Five to seven days after inoculation, pathogen growth was evaluated by measurement of the optical density (OD) at λ 405 nm with a TecanGENios Pro microplate reader (Ramsey, USA) (5 measurements per well using multiple reads in a 3 × 3 square). Each experiment was carried out in triplicates.

### Assays for anthelmintic activity

The Bristol N2 wild type strain of *Caenorhabditis elegans* was used in the anthelmintic assay. The nematodes were cultured on NGM (Nematode Growth Media) Petri plates using the uracil auxotroph *E. coli* strain OP50 as a food source according to the methods described before^44^. The anthelmintic assay was carried out following the method developed by Thomsen et al.^46^. Briefly, after 4 days of cultivation, the nematodes were transferred from the Petri plate to a 15 mL conical centrifuge tube by rinsing each plate twice with 2 mL of M9 buffer. The worm suspension was centrifuged for 1 min at 800 g. After the removal of the supernatant, the nematodes were washed again with 2 mL of M9 buffer under the same conditions and, depending on the number of individuals, re-suspended in 2 to 8 mL of M9 buffer. To this suspension, 10 µL of a penicillin-streptomycin-solution (10 mg/mL) was added. After triply counting the nematodes in 10 µL solution droplets under a stereo microscope (Olympus SZX12), the worm number was adjusted to 20–30 animals per 20 µL. The assay was performed in 384 well plates. The outer wells were filled with water to minimize evaporation. To the test wells, 20 µL of worm suspension was added and the number of living and dead animals in each well were counted using the cell culture microscope Olympus CKX41. The number of living nematodes was set as 100%. At staggered intervals, 20 µL test solution (test compound in 4% DMSO in M9 buffer) was added followed by a microscopic enumeration of living and dead test organisms after 30 min of incubation. For all test plates, the solvent DMSO (2%) and the standard anthelmintic drug ivermectin (10 µg/mL) were used as negative and positive controls, respectively.

### Statistical analysis

All the assays were done in triplicate. A one-way analysis of variance (ANOVA) was used to determine statistical significance, followed by a multiple comparison test (Tukey’s post hoc test) with a significance level of *p* < 0.05.

## Supplementary Information

Supplementary Information.

## Data Availability

Data are available upon request from the first author, N.Z.M.
