# Supplementary material for: Comparative metabolic profiling and quantitative analysis of metabolites in different tissues of Ajuga turkestanica by ESI-UHPLC-QqTOF-MS and NMR
Source: Sci Rep. 2024 Nov 15;14:28179. doi: 10.1038/s41598-024-71546-5 (PMC11568135; doi:10.1038/s41598-024-71546-5)
Supplement: Supplementary file 1 — Supplementary Information. [file 41598_2024_71546_MOESM1_ESM.pdf]

Comparative metabolic profiling and quantitative analysis of metabolites in different tissues of *Ajuga turkestanica* by ESI-UHPLC-QqTOF-MS and NMR

Nilufar Z. Mamadalieva[1-4*], Michal Šoral[5], Elana Kysil[4], Pauline Stark[4], Andrej Frolov[4], Ludger A. Wessjohann[4*]

[1] New Uzbekistan University, 100000 Tashkent, Movarounnahr Str 1, Mirzo-Ulug'bek district, Uzbekistan
[2] Institute of the Chemistry of Plant Substances, Uzbekistan Academy of Sciences, Tashkent 100170, M. Ulugbek Str 77, Uzbekistan
[3] Tashkent Institute of Irrigation and Agricultural Mechanization Engineers, National Research University, Tashkent 100000, Kori Niyazov Str. 39, Uzbekistan
[4] Department of Bioorganic Chemistry, Leibniz Institute of Plant Biochemistry, Weinberg 3, 06120 Halle (Saale), Germany
[5] Analytical Department, Institute of Chemistry, Slovak Academy of Sciences, Dúbravská cesta 9, SK-845 38 Bratislava, Slovak Republic

*email: wessjohann@ipb-halle.de, nmamadalieva@yahoo.com

Keywords: *Ajuga turkestanica*, UHPLC-MS, NMR, metabolite profiling, metabolite annotation, biological activity

List of Supporting Information

Fig. S1. QqTOF mass spectrum of 8-*O*-acetylharpagide (**7**) (TOF scan) acquired with TripleTOF MS/MS system in negative ion mode
Fig. S2. QqTOF mass spectrum of 6-deoxy-8-*O*-acetylharpagide (**11**) (TOF scan) acquired with TripleTOF MS/MS system in negative ion mode
Fig. S3a. QqTOF mass spectrum of turkesterone (**12**) (TOF scan) acquired with TripleTOF MS/MS system in negative ion mode
Fig. S3b. QqTOF mass spectrum of turkesterone (**12**) (TOF scan) acquired with TripleTOF MS/MS system in positive ion mode
Fig. S4a. QqTOF mass spectrum of 20-hydroxyecdysone (**17**) (TOF scan) acquired with TripleTOF MS/MS system in negative ion mode
Fig. S4b. QqTOF mass spectrum of 20-hydroxyecdysone (**17**) (TOF scan) acquired with TripleTOF MS/MS system in positive ion mode
Fig. S5. QqTOF mass spectrum of baicalin (**19**) (TOF scan) acquired with TripleTOF MS/MS system in negative ion mode
Fig. S6a. QqTOF mass spectrum of cyasterone (**25**) (TOF scan) acquired with TripleTOF MS/MS system in negative ion mode
Fig. S6b. QqTOF mass spectrum of cyasterone (**25**) (TOF scan) acquired with TripleTOF MS/MS system in positive ion mode
Fig. S7. QqTOF mass spectrum of cyasterone 22-acetate (**30**) (TOF scan) acquired with TripleTOF MS/MS system in negative ion mode
Fig. S8. QqTOF mass spectrum of 14-hydro-15-hydroxyajugapitin (**34**) (TOF scan) acquired with TripleTOF MS/MS system in positive ion mode
Fig. S9. QqTOF mass spectrum of chamaepitin (**35**) (TOF scan) acquired with TripleTOF MS/MS system in negative ion mode
Fig. S10. QqTOF mass spectrum of 14,15-dihydroajugachin A (ajubractin D) (**36**) (TOF scan) acquired with TripleTOF MS/MS system in positive ion mode
Fig. S11a. QqTOF mass spectrum of 14,15-dihydroajugachin B (**37**) (TOF scan) acquired with TripleTOF MS/MS system in negative ion mode
Fig. S11b. QqTOF mass spectrum of 14,15-dihydroajugachin B (**37**) (TOF scan) acquired with TripleTOF MS/MS system in positive ion mode
Fig. S12a. QqTOF mass spectrum of 14,15-dihydro-ajugapitin (**38**) (TOF scan) acquired with TripleTOF MS/MS system in negative ion mode
Fig. S12b. QqTOF mass spectrum of 14,15-dihydro-ajugapitin (**38**) (TOF scan) acquired with TripleTOF MS/MS system in positive ion mode
Fig. S13a. QqTOF mass spectrum of 14-hydro-15-methoxyajugachin B (**39**) (TOF scan) acquired with TripleTOF MS/MS system in positive ion mode

Fig. S13b. QqTOF mass spectrum of 14-hydro-15-methoxyajugachin B (**39**) (TOF scan) acquired with TripleTOF MS/MS system in negative ion mode

Fig. S14a. QqTOF mass spectrum of ajugachin B (**40**) (TOF scan) acquired with TripleTOF MS/MS system in positive ion mode

Fig. S14b. QqTOF mass spectrum of ajugachin B (**40**) (TOF scan) acquired with TripleTOF MS/MS system in negative ion mode

Fig. S15a. QqTOF mass spectrum of ajugapitin (**41**) (TOF scan) acquired with TripleTOF MS/MS system in positive ion mode

Fig. S15b. QqTOF mass spectrum of ajugapitin (**41**) (TOF scan) acquired with TripleTOF MS/MS system in negative ion mode

Fig. S16. QqTOF mass spectrum of ajuganipponin B (**42**) (TOF scan) acquired with TripleTOF MS/MS system in negative ion mode

Fig. S17. Antifungal activity of the methanolic extract of *Ajuga turkestanica* (AT)

Fig. S18. Cytotoxic activity of the methanolic extract of *Ajuga turkestanica* (AT) against HT29 and PC3 cells

Fig. S19. Anthelmintic activity methanolic extract of *Ajuga turkestanica* (AT)

Table S1. NMR signal assignment of ajugachin B (**40**) in MeOH-*d*4

Table S2. NMR signal assignment of 20-hydroxyecdysone (**17**) in MeOH-*d*4

Table S3. NMR signal assignment of *cis*-melilotoside (**5**) in MeOH-*d*4

Table S4. NMR signal assignment of 8-*O*-acetylharpagide (**7**) in MeOH-*d*4

Table S5. NMR signal assignment of harpagide (**4**) in MeOH-*d*4

Table S6. NMR signal assignment of sucrose (**3**) in MeOH-*d*4

Table S7. Approximate concentrations of compounds identified by 1D and 2D NMR in the methanolic extracts of different organs of *Ajuga turkestanica*

Supplementary material

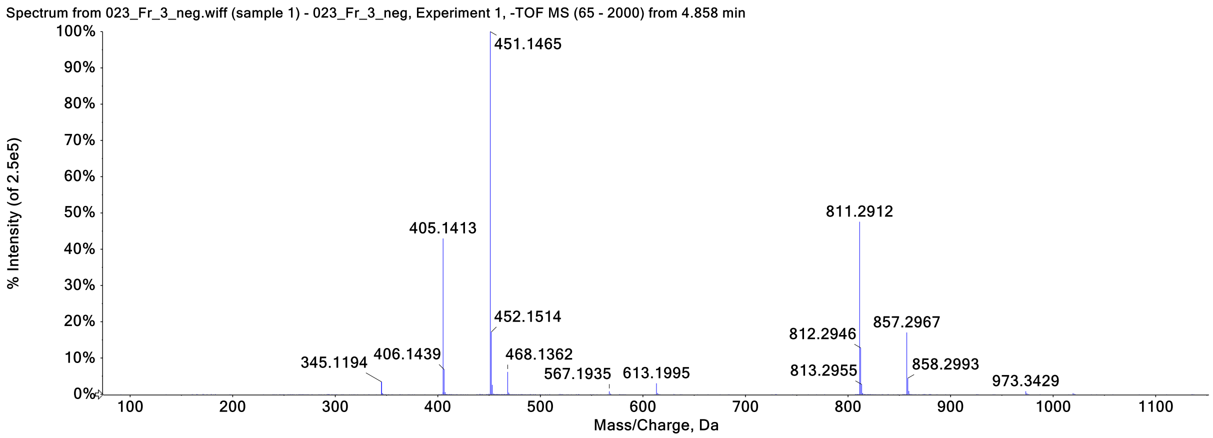

Fig. S1. QqTOF mass spectrum of 8-*O*-acetylharpagide (**7**) (TOF scan) acquired with TripleTOF MS/MS system in negative ion mode

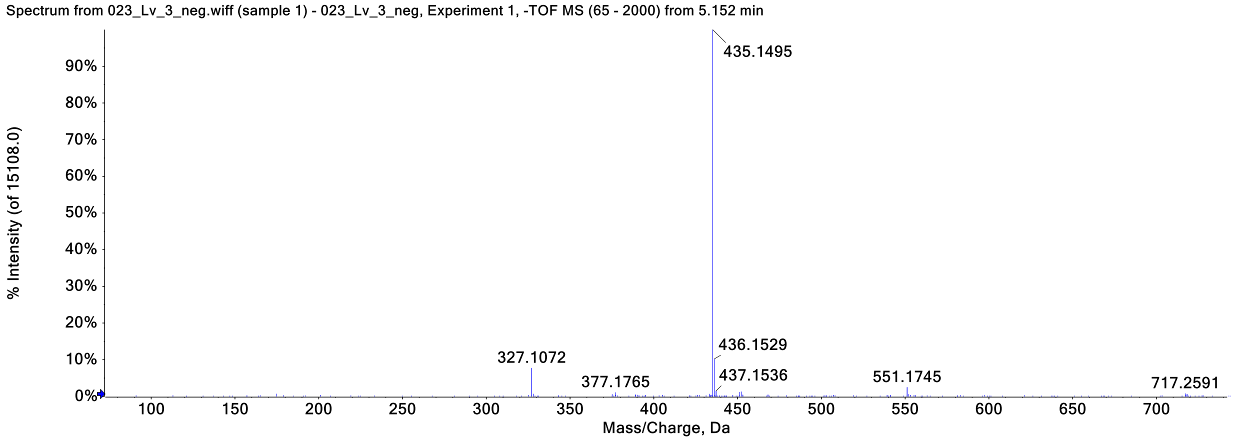

Fig. S2. QqTOF mass spectrum of 6-deoxy-8-*O*-acetylharpagide (**11**) (TOF scan) acquired with TripleTOF MS/MS system in negative ion mode

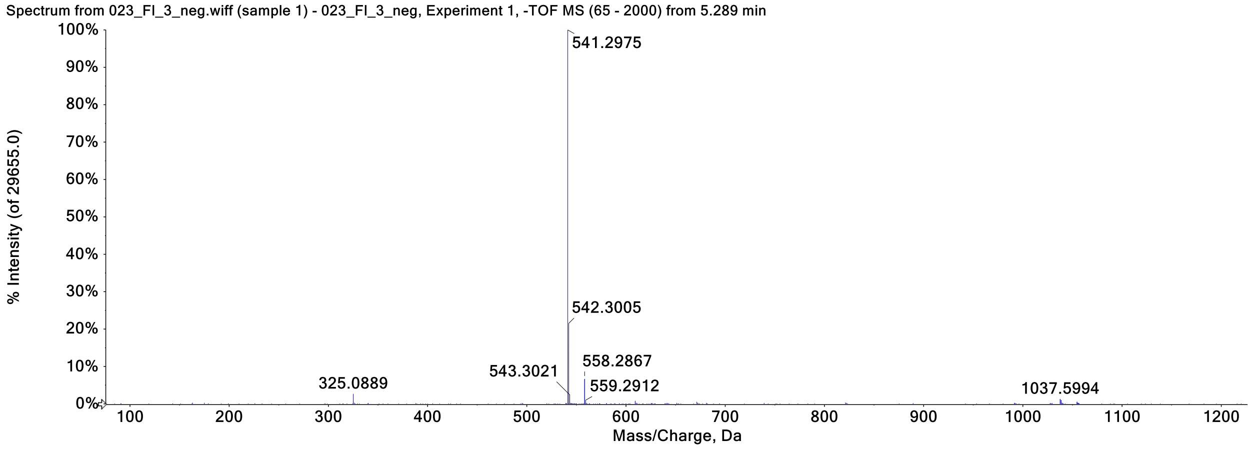

Fig. S3a. QqTOF mass spectrum of turkesterone (**12**) (TOF scan) acquired with TripleTOF MS/MS system in negative ion mode

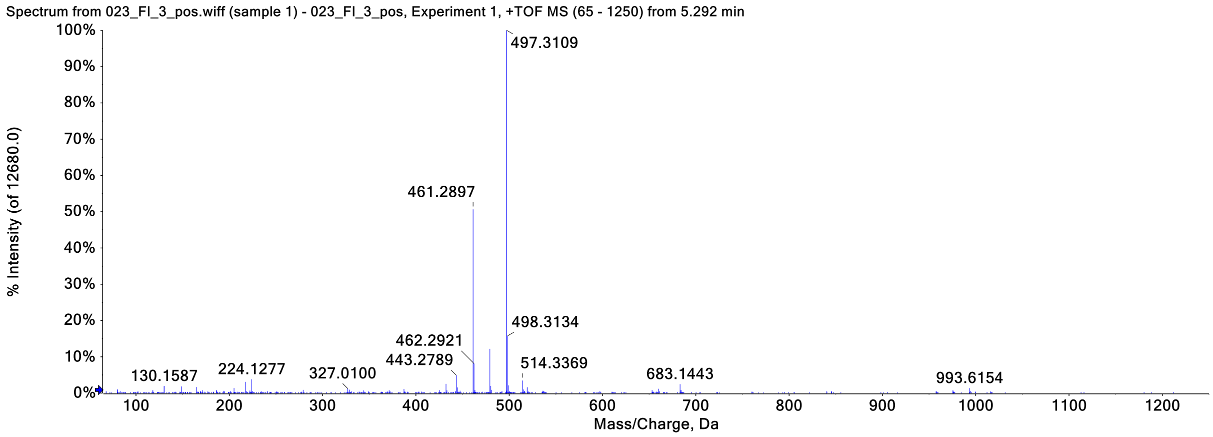

Fig. S3b. QqTOF mass spectrum of turkesterone (**12**) (TOF scan) acquired with TripleTOF MS/MS system in positive ion mode

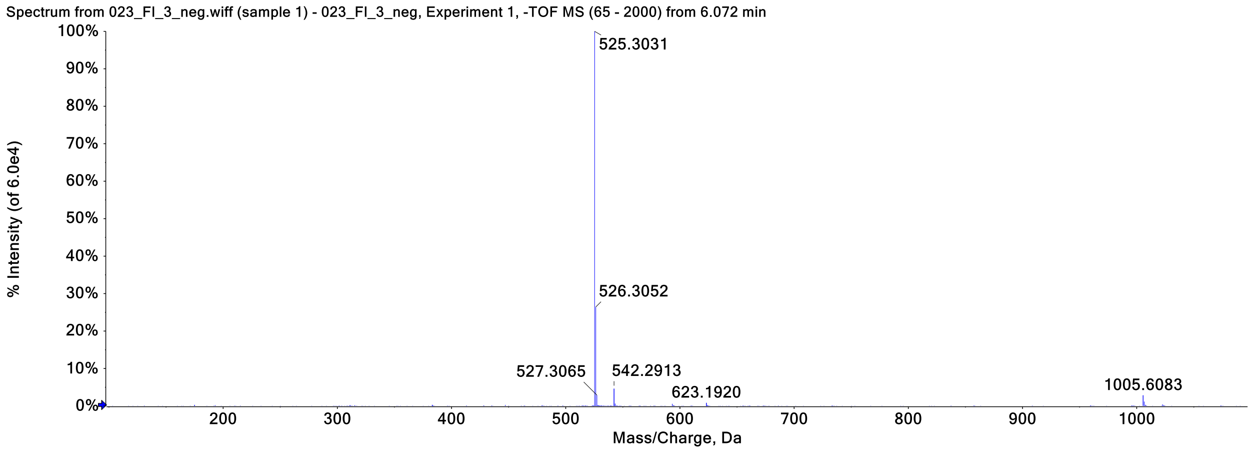

Fig. S4a. QqTOF mass spectrum of 20-hydroxyecdysone (**17**) (TOF scan) acquired with TripleTOF MS/MS system in negative ion mode

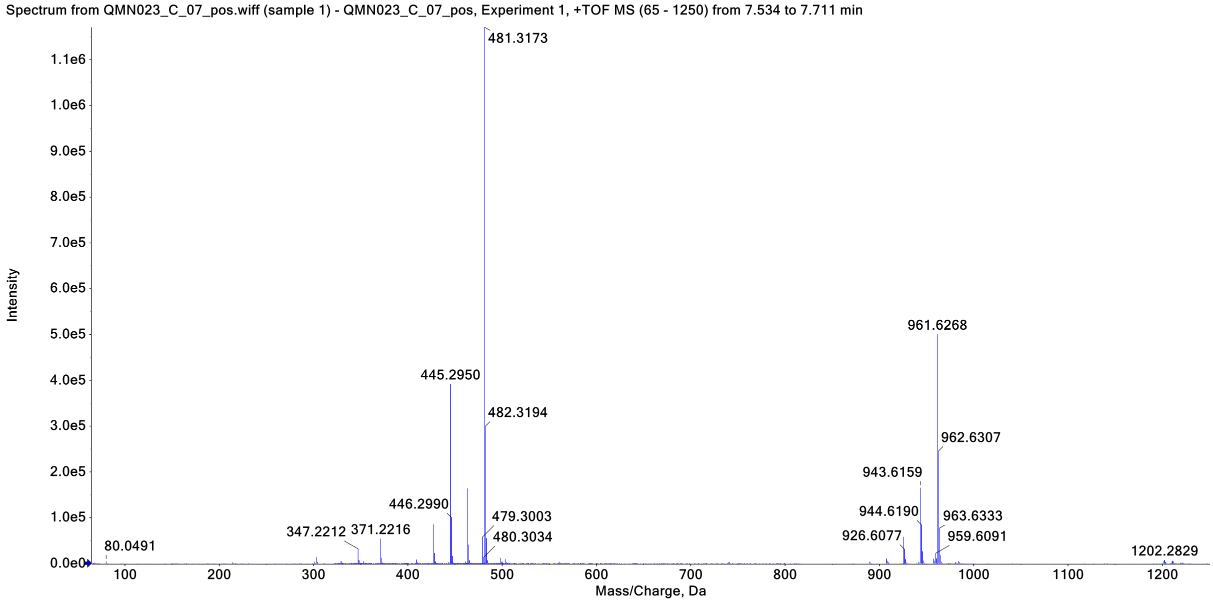

Fig. S4b. QqTOF mass spectrum of 20-hydroxyecdysone (**17**) (TOF scan) acquired with TripleTOF MS/MS system in positive ion mode

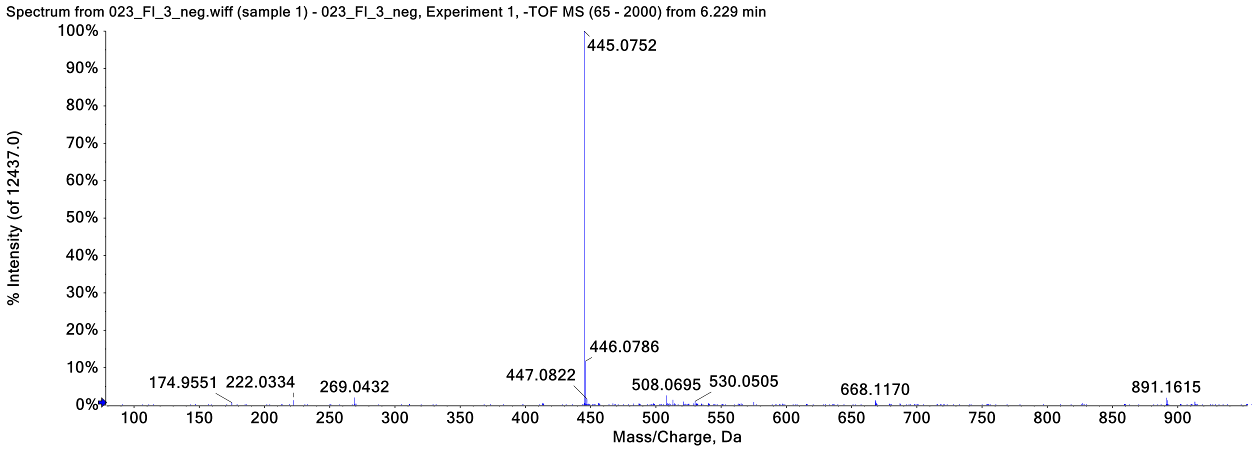

Fig. S5. QqTOF mass spectrum of baicalin (**19**) (TOF scan) acquired with TripleTOF MS/MS system in negative ion mode

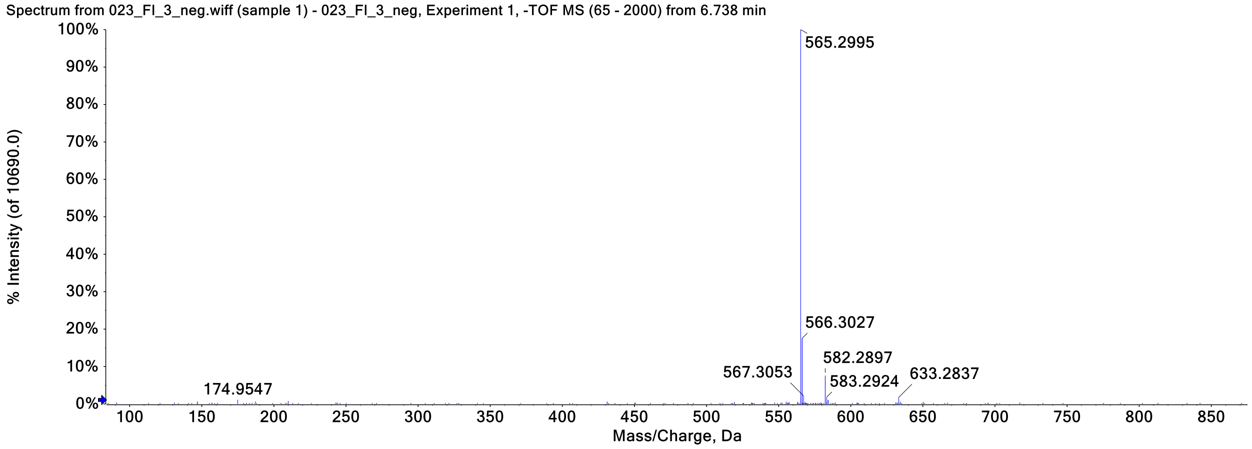

Fig. S6a. QqTOF mass spectrum of cyasterone (**25**) (TOF scan) acquired with TripleTOF MS/MS system in negative ion mode

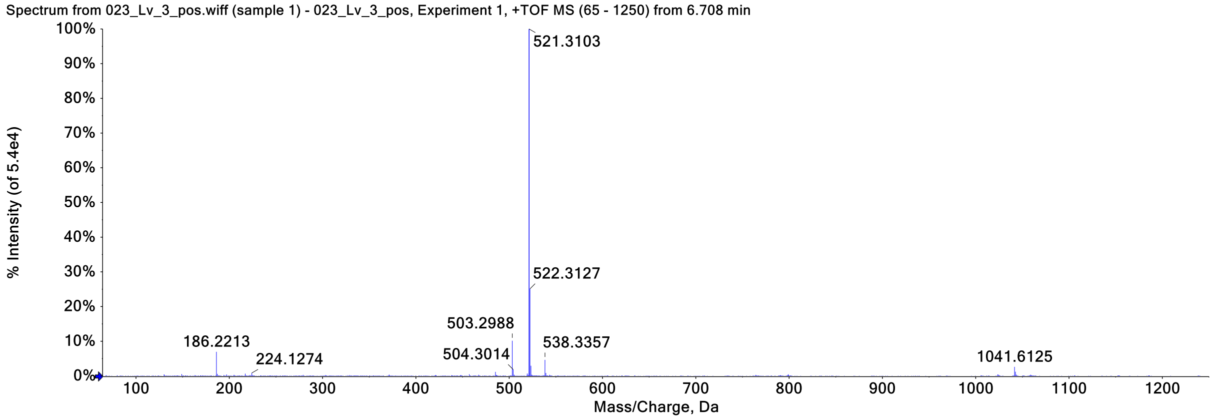

Fig. S6b. QqTOF mass spectrum of cyasterone (**25**) (TOF scan) acquired with TripleTOF MS/MS system in positive ion mode

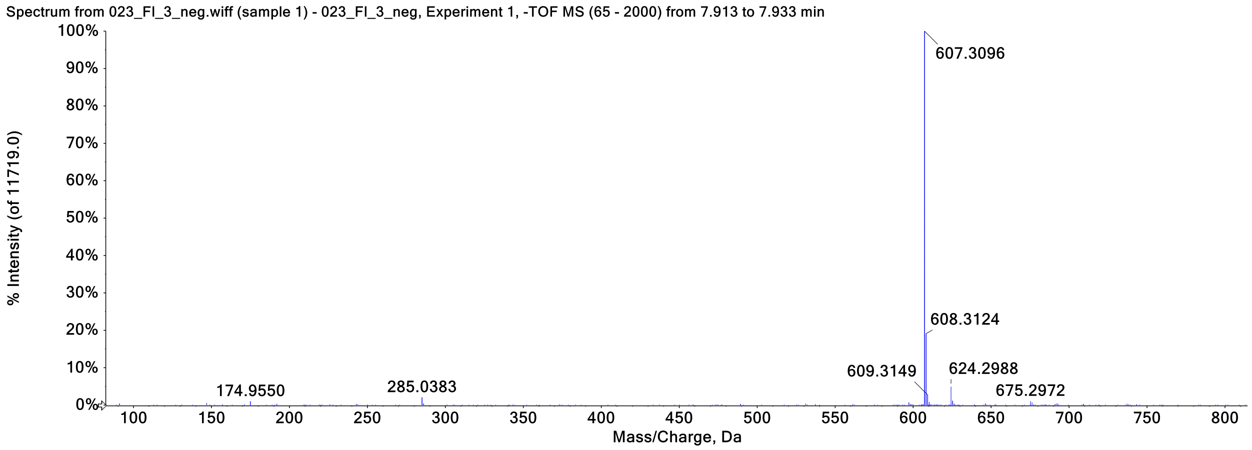

Fig. S7. QqTOF mass spectrum of cyasterone 22-acetate (**30**) (TOF scan) acquired with TripleTOF MS/MS system in negative ion mode

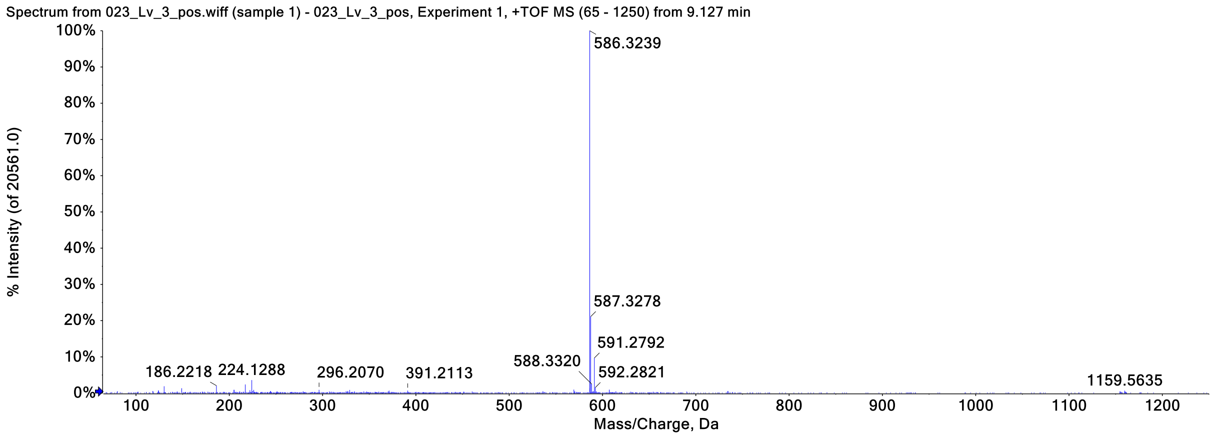

Fig. S8. QqTOF mass spectrum of 14-hydro-15-hydroxyajugapitin (**34**) (TOF scan) acquired with TripleTOF MS/MS system in positive ion mode

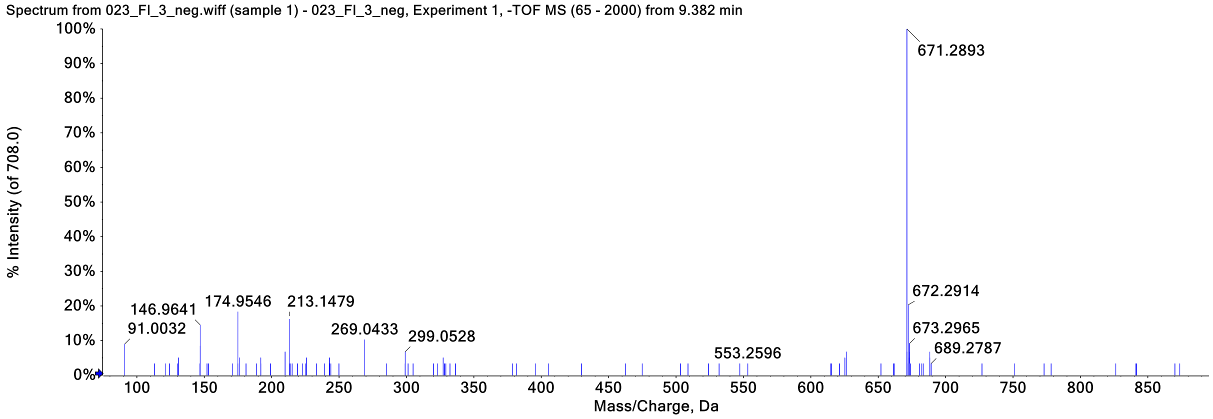

Fig. S9. QqTOF mass spectrum of chamaepitin (**35**) (TOF scan) acquired with TripleTOF MS/MS system in negative ion mode

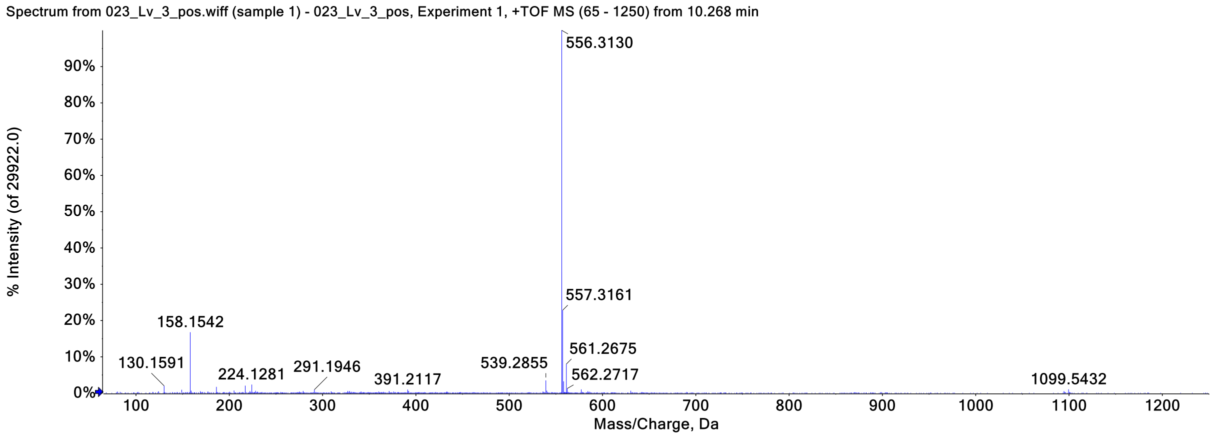

Fig. S10. QqTOF mass spectrum of 14,15-dihydroajugachin A (ajubractin D) (**36**) (TOF scan) acquired with TripleTOF MS/MS system in positive ion mode

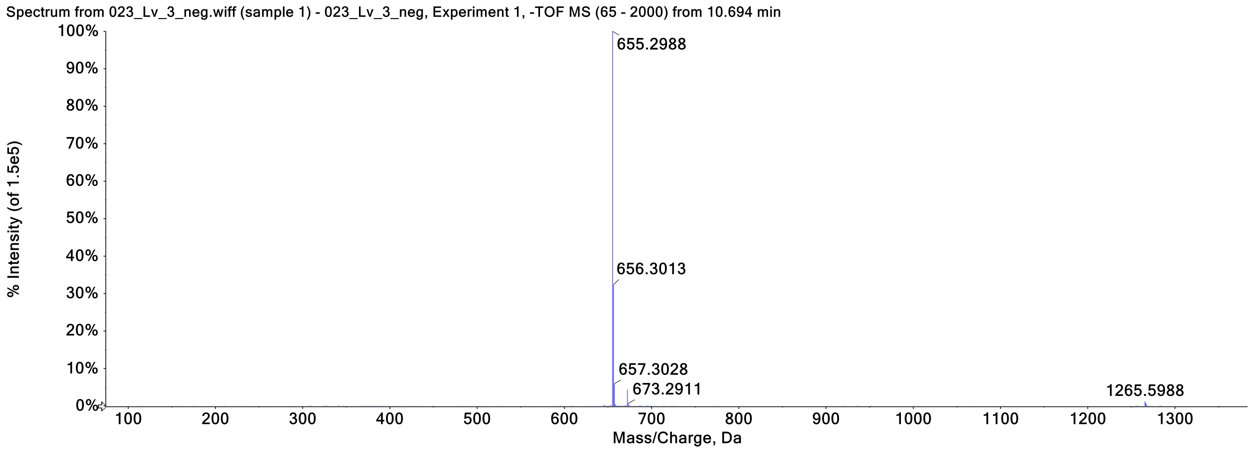

Fig. S11a. QqTOF mass spectrum of 14,15-dihydroajugachin B (**37**) (TOF scan) acquired with TripleTOF MS/MS system in negative ion mode

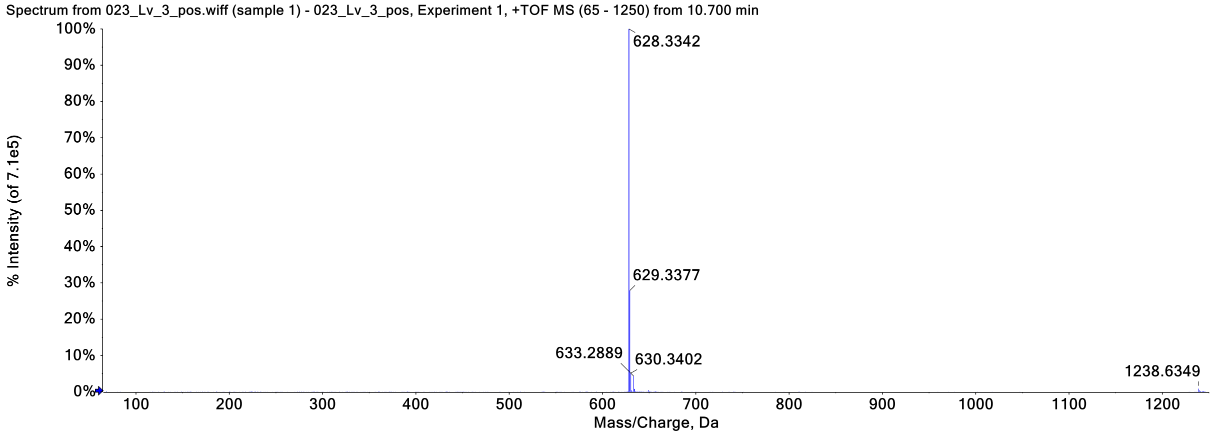

Fig. S11b. QqTOF mass spectrum of 14,15-dihydroajugachin B (**37**) (TOF scan) acquired with TripleTOF MS/MS system in positive ion mode

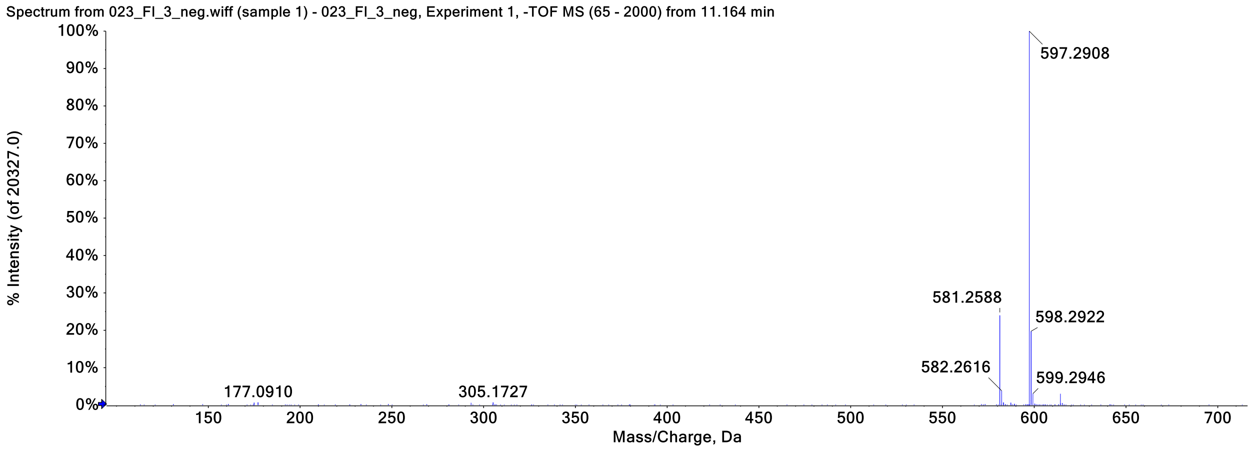

Fig. S12a. QqTOF mass spectrum of 14,15-dihydro-ajugapitin (**38**) (TOF scan) acquired with TripleTOF MS/MS system in negative ion mode

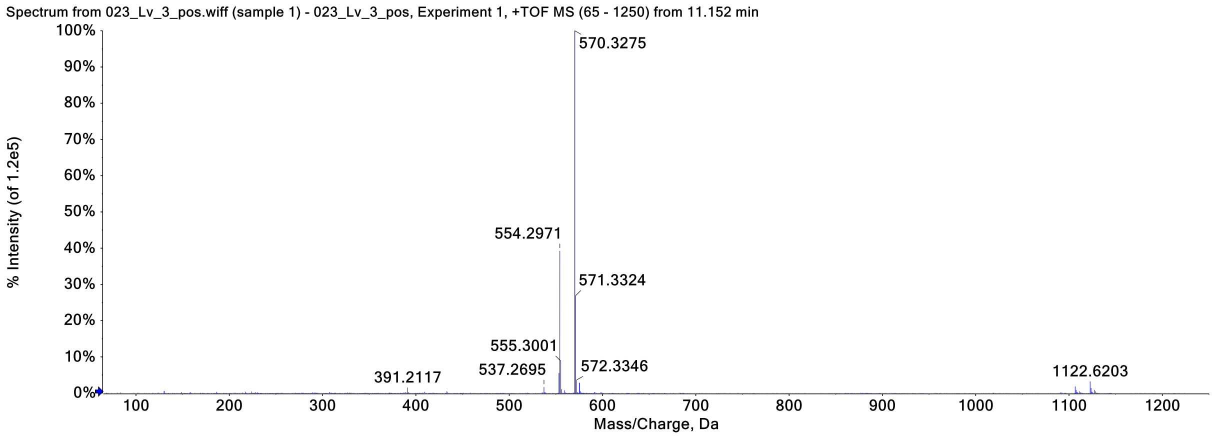

Fig. S12b. QqTOF mass spectrum of 14,15-dihydro-ajugapitin (**38**) (TOF scan) acquired with TripleTOF MS/MS system in positive ion mode

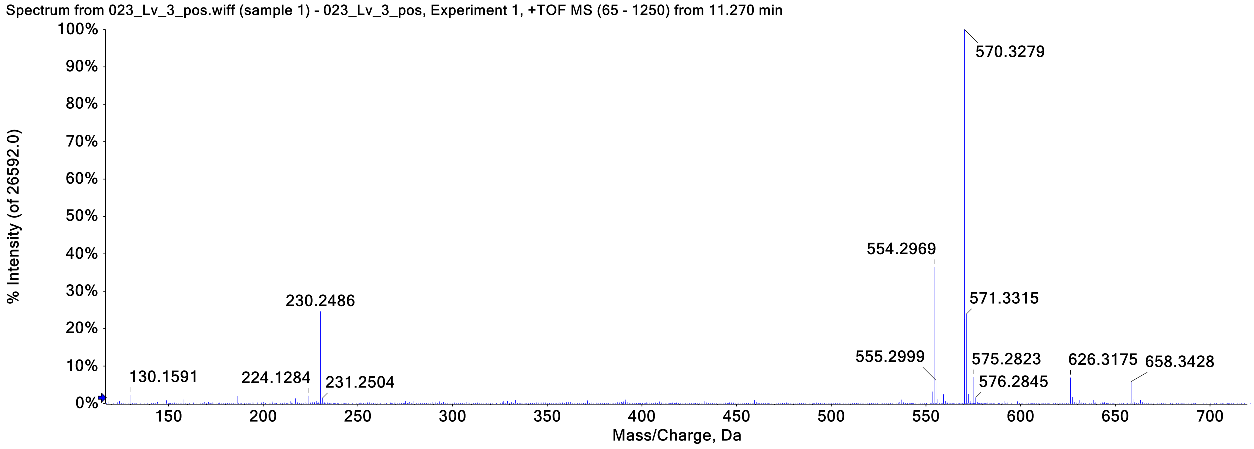

Fig. S13a. QqTOF mass spectrum of 14-hydro-15-methoxyajugachin B (**39**) (TOF scan) acquired with TripleTOF MS/MS system in positive ion mode

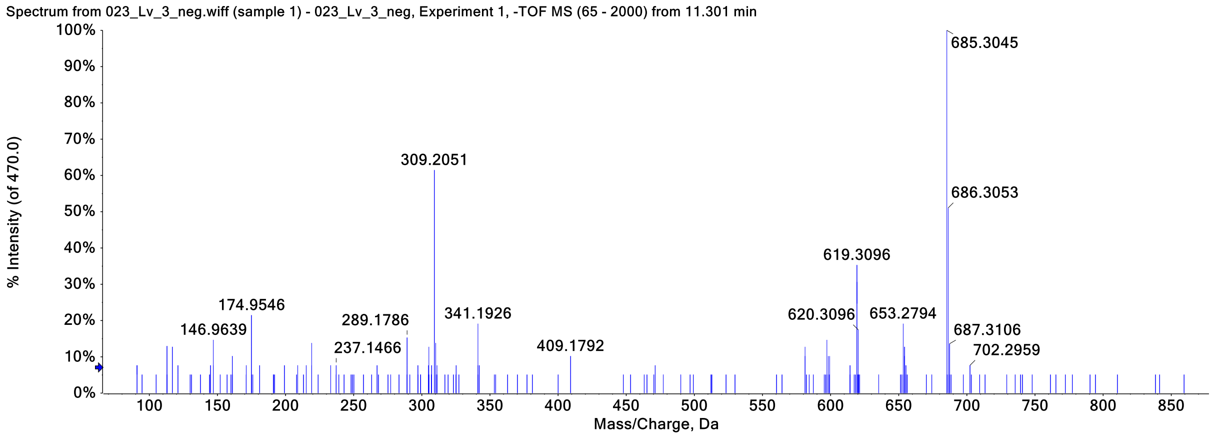

Fig. S13b. QqTOF mass spectrum of 14-hydro-15-methoxyajugachin B (**39**) (TOF scan) acquired with TripleTOF MS/MS system in negative ion mode

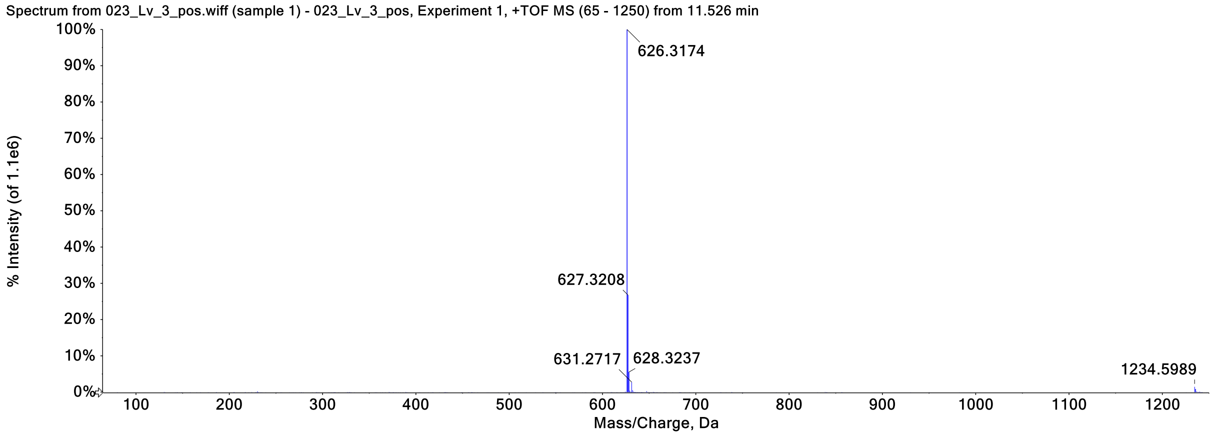

Fig. S14a. QqTOF mass spectrum of ajugachin B (**40**) (TOF scan) acquired with TripleTOF MS/MS system in positive ion mode

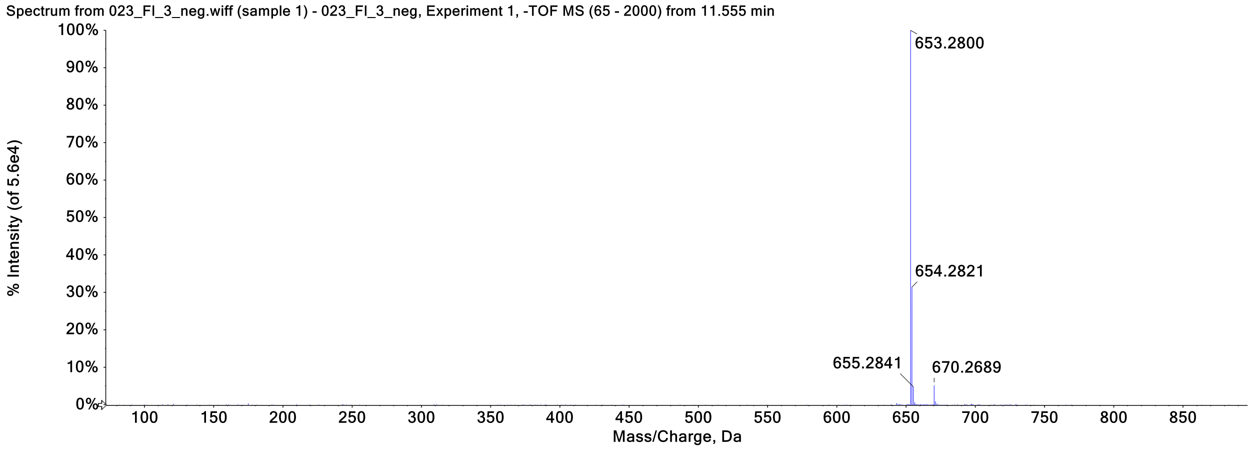

Fig. S14b. QqTOF mass spectrum of ajugachin B (**40**) (TOF scan) acquired with TripleTOF MS/MS system in negative ion mode

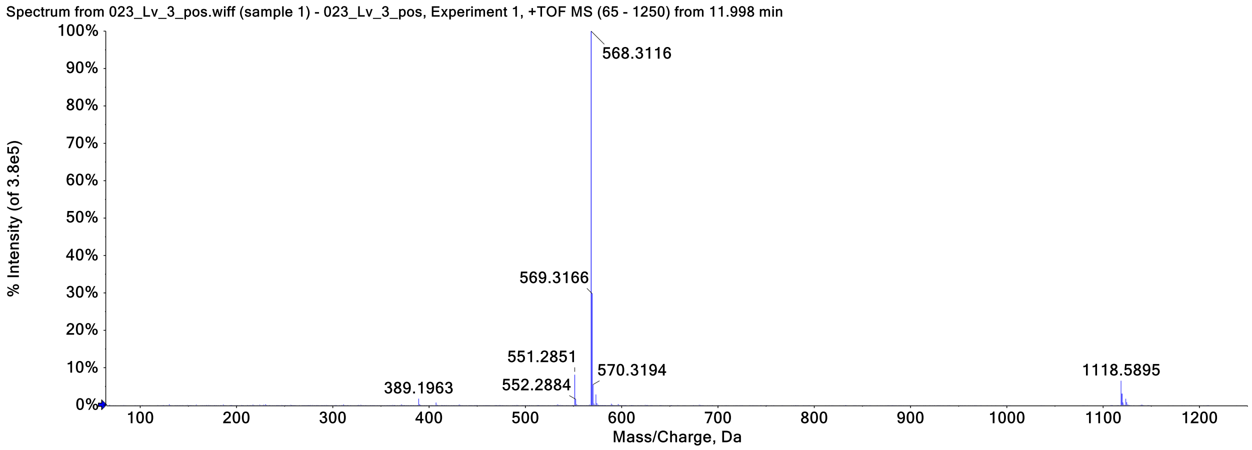

Fig. S15a. QqTOF mass spectrum of ajugapitin (**41**) (TOF scan) acquired with TripleTOF MS/MS system in positive ion mode

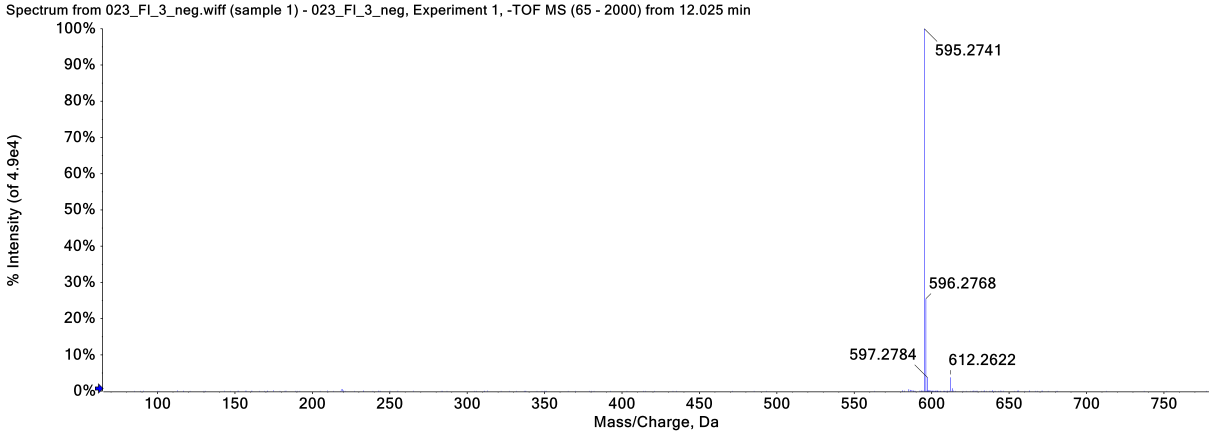

Fig. S15b. QqTOF mass spectrum of ajugapitin (**41**) (TOF scan) acquired with TripleTOF MS/MS system in negative ion mode

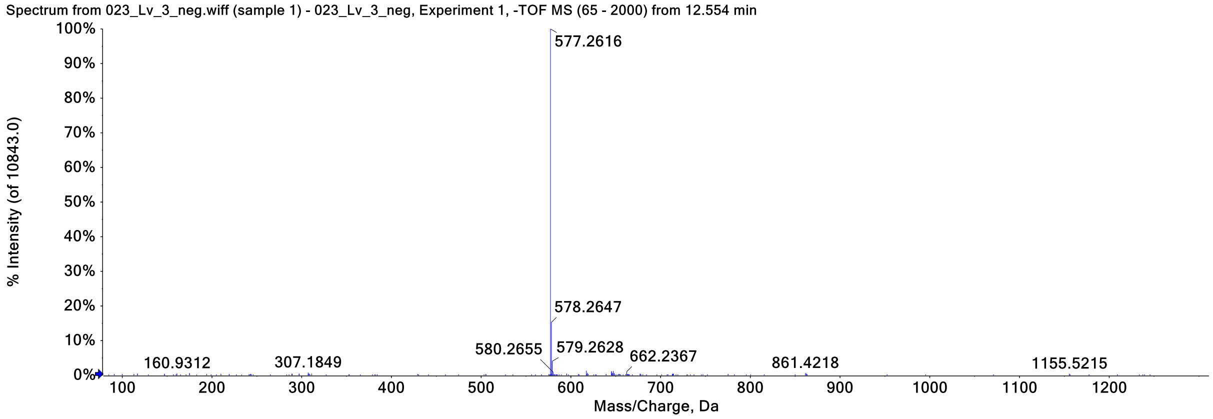

Fig. S16. QqTOF mass spectrum of ajuganipponin B (**42**) (TOF scan) acquired with TripleTOF MS/MS system in negative ion mode

A)

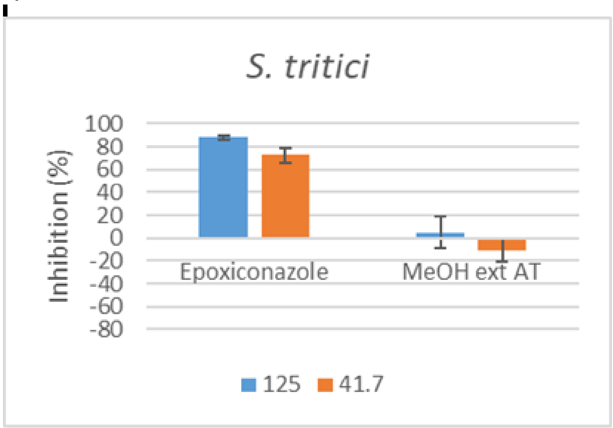

B)

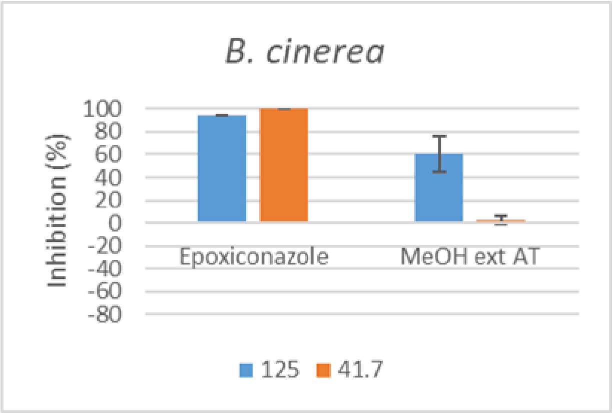

C)

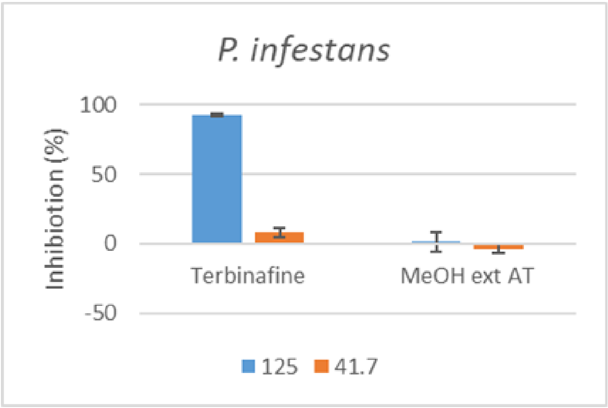

Fig. S17. Antifungal activity of the methanolic extract of *Ajuga turkestanica* (AT)

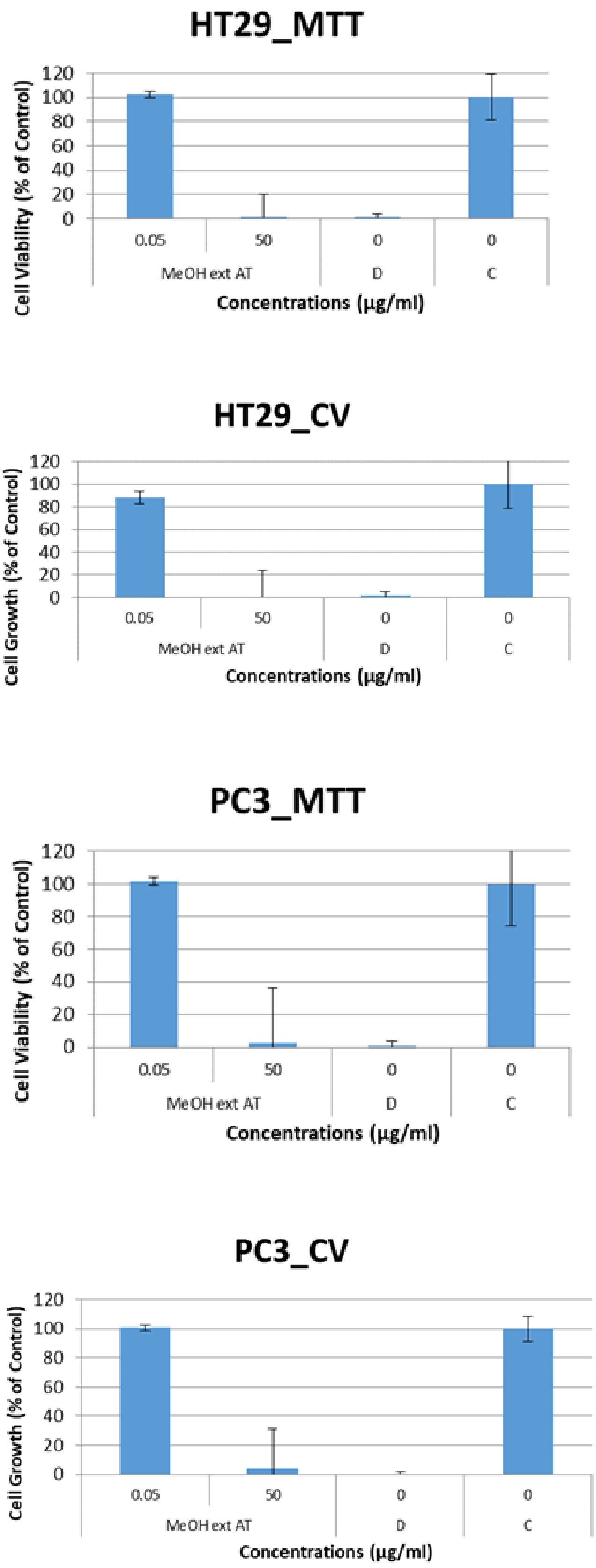

Fig. S18. Cytotoxic activity of the methanolic extract of *Ajuga turkestanica* (AT) against HT29 and PC3 cells. D-DMSO, C-control

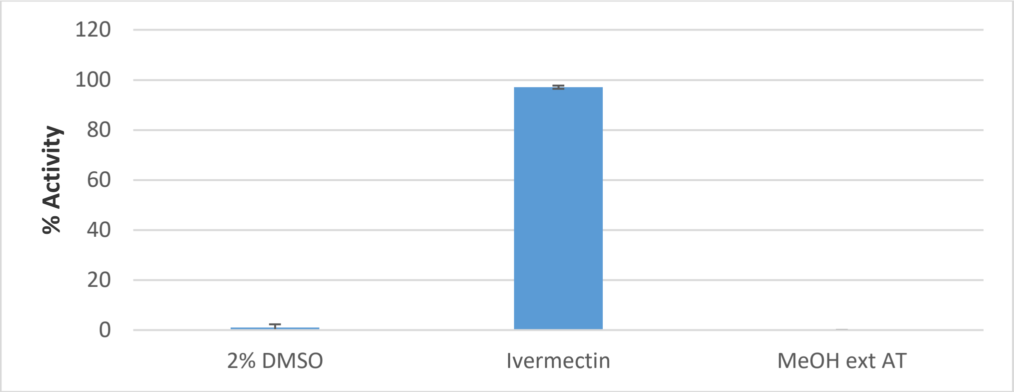

Fig. S19. Anthelmintic activity methanolic extract of *Ajuga turkestanica* (AT)

Table S1. NMR signal assignment of ajugachin B (**40**) in MeOH-d_4

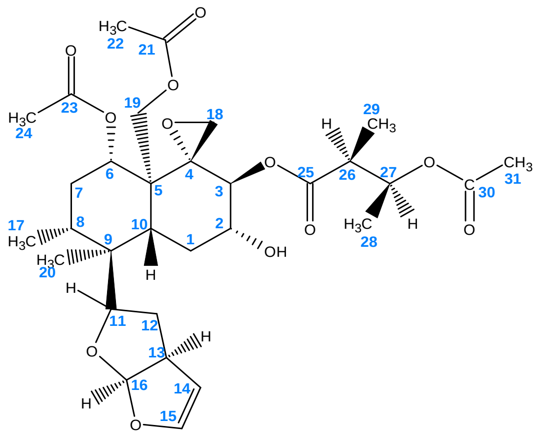

Position	^{1}H chemical shift / ppm	Multiplicity; J_{HH} / Hz	^{13}C chemical shift / ppm
1	1.74	m	31.8
	1.82	m	
2	3.63	m	71.6
3	5.14	d; 10.0	73.8
4	-	-	64.1
5	-	-	47.0
6	4.77	dd; 10.8, 4.9	72.5
7	1.44	m	34.2
	1.67	m	
8	1.53	m	37.2
9	-	-	41.1
10	1.84	m	44.6
11	4.03	m	85.9
12	1.75	m	32.0
	1.85	m	
13	3.61	m	47.4
14	4.87	m	103.3
15	6.49	dd; 1.9, 1.3	147.7
16	6.00	d; 6.2	109.1
17	0.85	d; 7.0	16.4
18	2.84	m	43.3
19	4.51	br d; 12.3	62.7
	4.70	d; 12.3	
20	0.96	s	14.3
21	-	-	172.9
22	2.07	s	21.1
23	-	-	171.5
24	1.89	s	21.2
25	-	-	174.6
26	2.68	dq; 8.4, 6.7	46.1

27	5.06	dq; 8.4, 7.1	72.8
28	1.18	d; 6.7	16.0
29	1.12	d; 7.1	12.9
30	-	-	172.1
31	1.99	s	21.3

Table S2. NMR signal assignment of 20-hydroxyecdysone (**17**) in MeOH-d_4

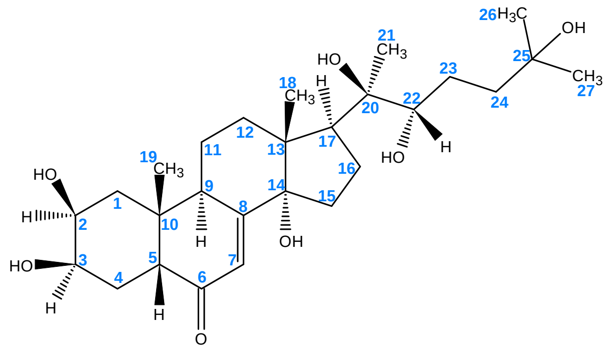

Position	^{1}H chemical shift / ppm	Multiplicity; J_{HH} / Hz	^{13}C chemical shift / ppm
1	1.43	m	37.3
	1.79	m	
2	3.84	m	n/a
3	3.94	m	n/a
4	1.74	m	32.9
5	2.38	m	51.7
6	-	-	206.6
7	5.80	d; 2.3	122.2
8	-	-	n/a
9	3.14	m	35.0
10	-	-	39.1
11	n/a	n/a	n/a
12	1.88	m	32.2
	2.10	m	
13	-	-	48.6
14	-	-	85.4
15	n/a	n/a	n/a
16	n/a	n/a	n/a
17	2.40	m	50.3
18	0.89	s	18.0
19	0.96	s	24.4
20	-	-	78.9
21	1.19	s	21.0
22	n/a	n/a	77.8
23	n/a	n/a	n/a
24	n/a	n/a	42.3
25	-	-	71.3
26	1.19	s	28.9
27	1.20	s	29.7

n/a = not available (impossible to assign unambiguously due to overlap or low signal to noise ratio)

Table S3. NMR signal assignment of *cis*-melilotoside (**5**) in MeOH-d_4

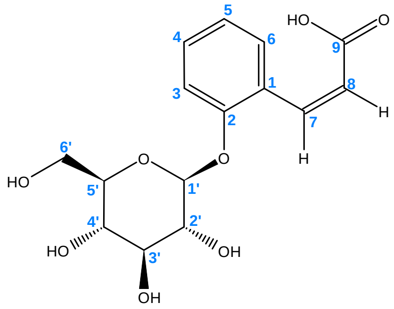

Position	^{1}H chemical shift / ppm	Multiplicity; J_{HH} / Hz	^{13}C chemical shift / ppm
1	-	-	127.8
2	-	-	156.2
3	7.14	dd; 8.3, 1.3	116.5
4	7.21	ddd; 8.3, 7.2, 1.7	130.2
5	6.94	ddd; 7.6, 7.2, 1.3	123.0
6	7.60	dd; 7.6, 1.7	131.0
7	6.90	d; 12.7	129.9
8	6.03	d; 12.7	127.1
9	-	-	175.2
1′	4.93	m	102.6
2′	3.50	m	74.9
3′	3.46	m	78.0
4′	3.40	m	71.2
5′	n/a	n/a	n/a
6′	3.69	m	62.6
	3.87	m	

n/a = not available (impossible to assign unambiguously due to overlap or low signal to noise ratio)

Table S4. NMR signal assignment of 8-*O*-acetylharpagide (**7**) in MeOH-d_4

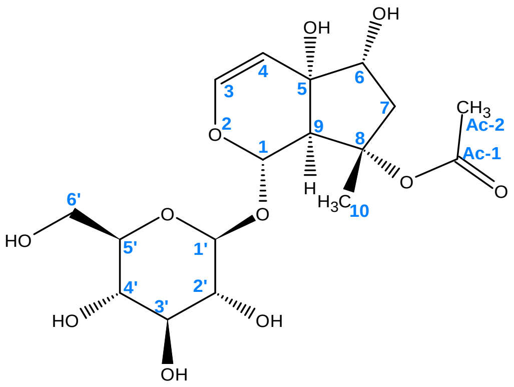

Position	^{1}H chemical shift / ppm	Multiplicity; J_{HH} / Hz	^{13}C chemical shift / ppm
1	6.07	d; 1.4	94.5
2	-	-	-
3	6.38	d; 6.4	143.9
4	4.91	d; 6.4	106.9
5	-	-	73.3
6	3.71	m	77.7
7	1.94	dd; 15.2, 4.5	45.9
	2.16	ddd; 15.2, 1.5, 1.1	
8	-	-	88.6
9	2.85	app s	55.4
10	1.45	s	22.5
1′	4.59	d; 7.9	99.9
2′	3.20	dd; 9.2, 7.9	74.5
3′	3.38	m	77.6
4′	3.29	m	71.6
5′	3.29	m	78.3
6′	3.69	m	62.8
	3.89	dd; 12.2, 2.0	
Ac-1	-	-	173.4
Ac-2	2.01	s	22.2

Table S5. NMR signal assignment of harpagide (**4**) in MeOH-d_4

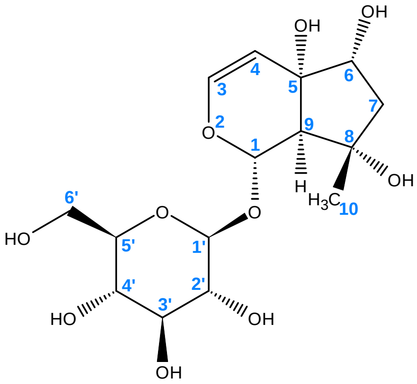

Position	[1]H chemical shift / ppm	Multiplicity; J_{HH} / Hz	[13]C chemical shift / ppm
1	5.73	d; 1.3	93.1
2	-	-	-
3	6.31	d; 6.4	142.5
4	4.94	dd; 6.4, 1.4	108.5
5	-	-	72.3
6	3.70	m	78.2
7	1.79	ddd; 13.2, 4.0	47.2
	1.90	m	
8	-	-	78.1
9	2.53	app s	59.5
10	1.24	s	24.9
1′	4.57	d; 7.9	99.2
2′	3.20	m	74.6
3′	n/a	n/a	n/a
4′	n/a	n/a	n/a
5′	n/a	n/a	n/a
6′	n/a	n/a	n/a
	n/a	n/a	

n/a = not available (impossible to assign unambiguously due to overlap or low signal to noise ratio)

Table S6. NMR signal assignment of sucrose (**3**) in MeOH-d_4

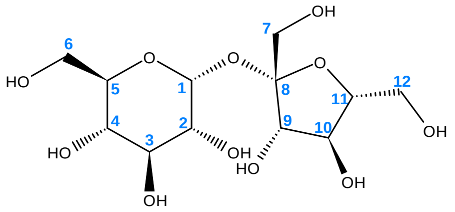

Position	^{1}H chemical shift / ppm	Multiplicity; J_{HH} / Hz	^{13}C chemical shift / ppm
1	5.38	d; 3.8	93.6
2	3.41	dd; 9.8, 3.8	73.2
3	3.70	t; 9.8	74.5
4	3.35	dd; 9.8, 9.0	71.3
5	3.82	m	74.4
6	3.71	m	62.1
	3.80	m	
7	3.59	d; 12.3	64.0
	3.63	d; 12.3	
8	-	-	105.2
9	4.10	m	79.2
10	4.02	m	75.6
11	3.75	m	83.7
12	3.75	m	63.3

Table S7. Approximate concentrations of compounds identified by 1D and 2D NMR in the methanolic extracts of different organs of *Ajuga turkestanica*

Extract	Compound											
	8-*O*-Acetylharpagide (**7**)		Harpagide (**4**)		Ajugachin B (**40**)		*cis*-Melilotoside (**5**)		Sucrose (**3**)		20-Hydroxyecdysone (**17**)	
	^{1}H atom number (δ_H / ppm)	*c* / mmol.dm^{-3}	^{1}H atom number (δ_H / ppm)	*c* / mmol.dm^{-3}	^{1}H atom number (δ_H / ppm)	*c* / mmol.dm^{-3}	^{1}H atom number (δ_H / ppm)	*c* / mmol.dm^{-3}	^{1}H atom number (δ_H / ppm)	*c* / mmol.dm^{-3}	^{1}H atom number (δ_H / ppm)	*c* / mmol.dm^{-3}
Leaves	3 (6.38)<br>Ac-2 (2.01)<br>7 (2.16)	4.3	3 (6.31)<br>10 (1.24)<br>1 (5.73)	1.9	16 (6.00)<br>31 (1.99)<br>29 (1.12)	0.7	7 (6.90)<br>8 (6.03)	2.7	9 (4.10)<br>1 (5.38)	3.5	7 (5.81)	0.54
Stems	3 (6.38)<br>Ac-2 (2.01)<br>1 (6.07)	11.6	3 (6.31)<br>10 (1.24)<br>1 (5.73)	1.1	-	<LoD or overlapped	7 (6.83)*<br>6 (7.62)*<br>4 (7.19)*	0.2	1 (5.38)	7.3	7 (5.81)	0.4
Roots	3 (6.38)<br>Ac-2 (2.01)<br>1 (6.07)	0.8	1 (5.73)	0.02	-	<LoD	8 (6.03)	0.04	9 (4.10)<br>1 (5.38)	0.8	-	<LoD
Seeds	3 (6.38)<br>Ac-2 (2.01)<br>7 (2.16)	1.7	3 (6.31)<br>10 (1.24)<br>1 (5.73)	3.1	31 (1.99)	0.04	8 (6.03)	0.01	-	<LoD or overlapped	-	<LoD or overlapped
Fruits	3 (6.38)<br>Ac-2 (2.01)<br>7 (2.16)	3.7	3 (6.31)<br>10 (1.24)<br>1 (5.73)	2.6	16 (6.00)<br>31 (1.99)<br>29 (1.12)	0.3	7 (6.90)<br>6 (7.60)<br>8 (6.03)	0.3	9 (4.10)<br>1 (5.38)	1.3	7 (5.81)	0.6

Flowers	3 (6.38)<br>Ac-2 (2.01)<br>7 (2.16)	0.5	3 (6.31)<br>10 (1.24)<br>1 (5.73)	0.6	16 (6.00)<br>31 (1.99)	0.1	6 (7.60)<br>8 (6.03)	0.05	9 (4.10)<br>1 (5.38)	0.3	-	<LoD or overlapped

*a dramatic change in the ^{1}H chemical shift was noticed, possibly due to the different degree of ionization of the carboxylic acid group; no buffer was used; LoD = Limit of detection
